# Loss of Hepatic Angiotensinogen Attenuates Diastolic Dysfunction in Heart Failure with Preserved Ejection Fraction

**DOI:** 10.1002/advs.202507554

**Published:** 2025-09-01

**Authors:** Zetao Heng, Min Hong, Zhaocai Zhang, Ximing Ye, Jianan Zhou, Wentao Wu, Jiabing Rong, Yinchuan Xu

**Affiliations:** ^1^ Department of Cardiology, The Second Affiliated Hospital, School of Medicine Zhejiang University Hangzhou 310009 China; ^2^ State Key Laboratory of Transvascular Implantation Devices Hangzhou 310009 China; ^3^ Heart Regeneration and Repair Key Laboratory of Zhejiang province Hangzhou 310009 China; ^4^ Transvascular Implantation Devices Research Institute Hangzhou 310053 China; ^5^ Binjiang Institute of Zhejiang University Hangzhou 310053 China; ^6^ Department of Critical Care Medicine, The Second Affiliated Hospital, School of Medicine Zhejiang University Hangzhou 310009 China

**Keywords:** 18β‐glycyrrhetinic acid, angiotensin II‐independent mechanism, angiotensinogen, heart failure with preserved ejection fraction, microvascular angiogenesis

## Abstract

Heart failure with preserved ejection fraction (HFpEF) is a prevalent complex syndrome characterized by diastolic dysfunction with limited therapeutic options. While the renin‐angiotensin system (RAS) is implicated in heart failure pathogenesis, the causal contribution of angiotensinogen (AGT), the unique precursor of the RAS, to HFpEF remains undefined. Using a two‐hits mouse HFpEF model (high‐fat diet + L‐NAME), consistent upregulation of hepatic and plasma AGT  is identified in wild‐type mice of both sexes. Critically, hepatocyte‐specific AGT deletion directly ameliorated diastolic dysfunction in male and female HFpEF mice, whereas systemic angiotensin II blockade (losartan) failed to improve cardiac diastolic function. Mechanistically, hepatic AGT drove HFpEF through LRP2‐mediated internalization in cardiac endothelial cells, suppressing the GATA2/Pim3 signaling axis, which inhibited microvascular angiogenesis and ultimately exacerbated diastolic dysfunction. To validate therapeutic potential, it is demonstrated that 18β‐glycyrrhetinic acid – identified as a potent hepatic AGT inhibitor – significantly improved cardiac diastolic function in HFpEF mice. These findings establish hepatic AGT as a causal contributor to HFpEF pathogenesis and reveal its therapeutic targeting as a promising strategy.

## Graphical abstract

Increased hepatic angiotensinogen (AGT) abundance leads to cardiac diastolic dysfunction via the AngII‐independent pathway. Liver‐derived AGT is internalized by LRP2 in cardiac endothelial cells, subsequently contributing to myocardial diastolic dysfunction by suppressing microvascular angiogenesis via inhibiting the GATA2/Pim3 pathway. In vivo 18β‐glycyrrhetinic acid infusion may be a promising approach targeting hepatic AGT for heart failure with preserved ejection fraction (HFpEF) therapy.

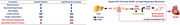

## 1 Introduction

Heart failure (HF), a leading cause of global mortality,^[^ ^1^, ^2^ ^]^ is classified by left ventricular ejection fraction (LVEF) into three subtypes: HF with reduced EF (HFrEF, LVEF ≤ 40%), HF with mildly reduced EF (HFmrEF, LVEF 41–49%), and HF with preserved EF (HFpEF, LVEF ≥ 50%).^[^ ^3^, ^4^, ^5^ ^]^ HFpEF is the predominant form in adults aged ≥ 65 years,^[^ ^6^ ^]^ disproportionately affecting women and the elderly, and exhibits strong epidemiological associations with obesity, metabolic syndrome, insulin resistance, and atrial fibrillation.^[^ ^7^ ^]^ Despite its high prevalence, evidence‐based pharmacotherapies remain limited. Only SGLT2 inhibitors^[^ ^8^ ^]^ and finerenone^[^ ^9^, ^10^ ^]^ have demonstrated clinical benefits in reducing adverse cardiovascular outcomes. This underscores the critical need for novel HFpEF therapeutics.

The renin‐angiotensin system (RAS) plays a key role in regulating blood pressure and fluid homeostasis and is implicated in HF pathophysiology.^[^ ^11^ ^]^ While RAS inhibitors, including angiotensin‐converting enzyme inhibitors (ACEIs), angiotensin II receptor blockers (ARBs), and mineralocorticoid receptor antagonists (MRAs) are therapeutic cornerstones for HFrEF,^[^ ^5^, ^12^, ^13^, ^14^, ^15^ ^]^ large‐scale clinical trials consistently show no mortality benefit from conventional RAS inhibition in HFpEF,^[^ ^16^, ^17^, ^18^ ^]^ implying distinct disease mechanisms. Mechanistic studies further reveal divergent RAS activation patterns between HF subtypes. HFrEF exhibits elevated plasma renin/aldosterone strongly correlated with adverse outcomes.^[^ ^19^, ^20^, ^21^ ^]^ In contrast, HFpEF shows minimal dynamic changes in these RAS components, with no demonstrable association between renin/aldosterone levels and diastolic dysfunction.^[^ ^22^, ^23^ ^]^ This raises the question of whether RAS activation plays a causal role in HFpEF pathogenesis.

Angiotensinogen (AGT) is the sole precursor for all angiotensin peptides. Conventional RAS inhibitors (e.g., ACEIs and ARBs), which systemically target AngII and are taken daily, have not shown consistent beneficial effects in HFpEF. Recently, AGT has emerged as a potential mediator in HFpEF. A cross‐sectional study reported a significant correlation between plasma AGT levels and impaired cardiac diastolic function.^[^ ^24^, ^25^ ^]^ After adjusting for blood pressure, left ventricular structural parameters (e.g., wall thickness), and other confounders, plasma AGT concentrations remained independently correlated with the E/e’ ratio – a validated marker of elevated left ventricular filling pressure – unlike other classical RAS components.^[^ ^26^ ^]^ While this association suggests AGT may contribute to diastolic impairment through a non‐canonical pathway, direct causal evidence remains absent.

Therefore, the current study aims to: 1) investigate the pathogenic role of AGT in HFpEF progression, and 2) elucidate the molecular mechanisms by which AGT promotes diastolic dysfunction. Addressing these knowledge gaps may identify AGT as a promising therapeutic target beyond the RAS axis, offering new avenues for HFpEF treatment.

## 2 Results

### 2.1 HFpEF increased AGT Abundances in Liver and Plasma

**Figure 1:**
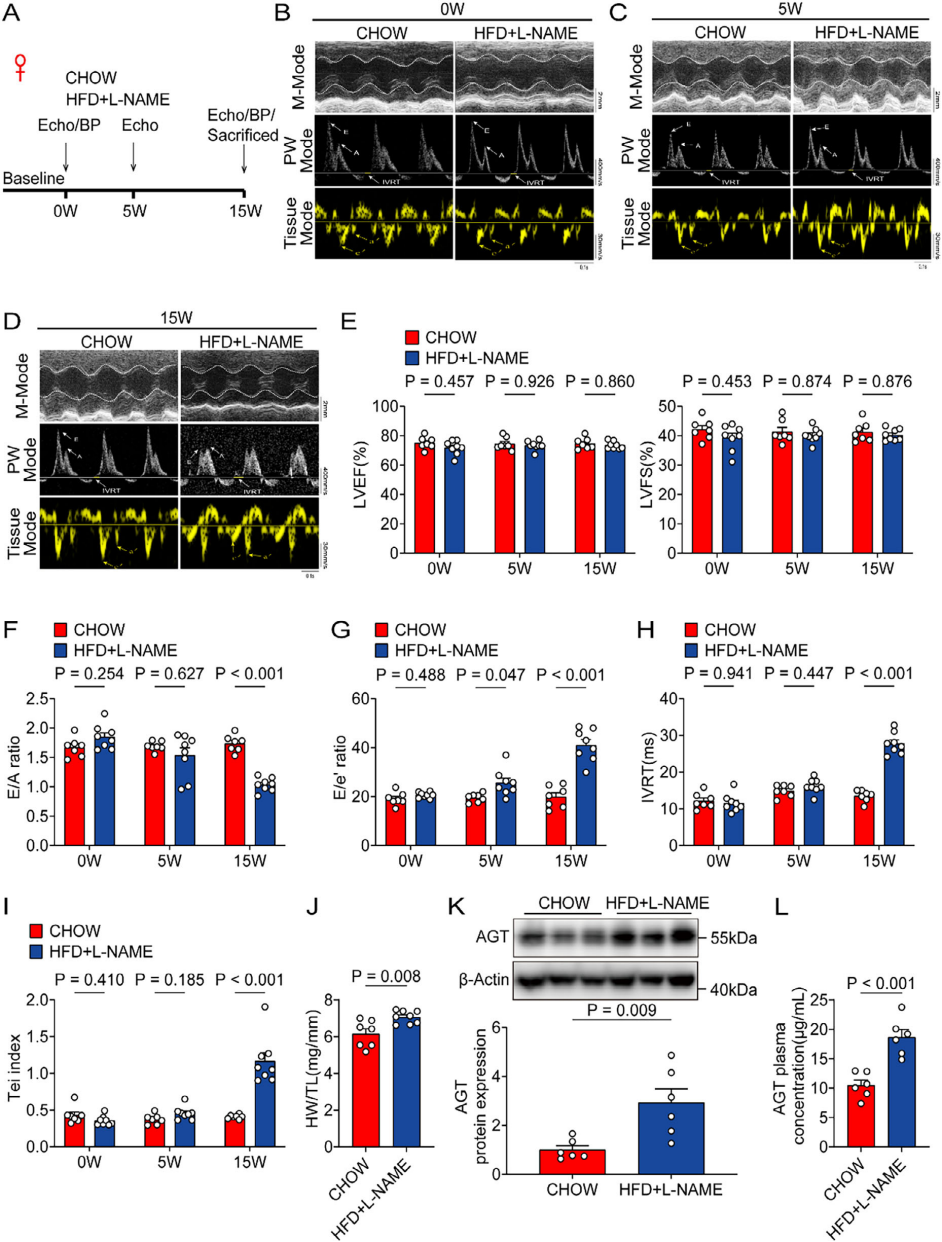
HFpEF increased hepatic AGT abundances and plasma AGT concentrations in female mice. A) Experimental workflow and analysis for HFpEF model establishment in female mice (n = 7 for CHOW group, and n = 8 for HFD+L‐NAME group). B) Representative echocardiography images obtained from female mice at baseline (n = 7 for the CHOW group, and n = 8 for the HFD+L‐NAME group). C) Representative echocardiography images obtained from female mice fed with a high‐fat diet and L‐NAME for 5 weeks (n = 7 for CHOW group, and n = 8 for HFD+L‐NAME group). D) Representative echocardiography images obtained from female mice fed with a high‐fat diet and L‐NAME for 15 weeks (n = 7 for CHOW group, and n = 8 for HFD+L‐NAME group). E) Left ventricular ejection fraction (LVEF%) and left ventricular fraction shortening (LVFS%) were quantified via echocardiography (n = 7 for the CHOW group, and n = 8 for the HFD+L‐NAME group). F) E/A ratio was quantified via echocardiography (n = 7 for the CHOW group, and n = 8 for the HFD+L‐NAME group). G) E/e’ ratio was quantified via echocardiography (n = 7 for the CHOW group, and n = 8 for the HFD+L‐NAME group). H) IVRT was quantified via echocardiography (n = 7 for the CHOW group, and n = 8 for the HFD+L‐NAME group). I) Tei index was quantified via echocardiography (n = 7 for the CHOW group, and n = 8 for the HFD+L‐NAME group). J) Ratio of heart weight to tibia length (HW/TL) was measured in female mice 15 weeks after feeding with HFD+L‐NAME (n = 7 for CHOW group, and n = 8 for HFD+L‐NAME group). K) Western blotting detection and quantification of AGT protein abundances in livers obtained from female mice 15 weeks after feeding with HFD and L‐NAME (n = 6 for each group). L) Plasma AGT concentrations in female mice fed with HFD and L‐NAME for 15 weeks were measured by ELISA (n = 6 for each group). Two‐way ANOVA was used for statistical analysis in E–I, and Student's *t* test was used for statistical analysis in J–L.

To determine the association of AGT and HFpEF, mice were fed with a high‐fat diet (HFD) and N^[ω]^‐Nitro‐L‐arginine methyl ester hydrochloride (L‐NAME) to induce HFpEF.^[^ ^27^ ^]^ Cardiac function was assessed by echocardiography at selected intervals (baseline, 5 and 15 weeks) after HFD+L‐NAME challenge (**Figure** **1**A; Figure S1A, Supporting Information). HFD+L‐NAME treatment resulted in increased heart weight (Figure 1J) and significant myocardial diastolic dysfunction in female mice after 15 weeks, as shown by decreased E/A ratio, increased E/e’ ratio, IVRT, and left ventricular Tei index (Figure 1B–D, F–I). However, myocardial systolic function remained unchanged after treatment (Figure 1E), suggesting that “two‐hits” stimulation, HFD plus L‐NAME, could successfully establish HFpEF model in female mice. Consistently, HFD+L‐NAME treatment could also induce similar myocardial diastolic dysfunction and increase heart weight in male mice after 15 weeks (Figure S1B–J, Supporting Information).

Since the liver is the main organ for the generation of AGT,^[^ ^28^ ^]^ we measured hepatic AGT expression after HFpEF model establishment. Western blotting revealed an increase in AGT protein abundances in liver tissues 15 weeks after HFD+L‐NAME treatment in both female and male mice (Figure 1K; Figure S1K, Supporting Information). Furthermore, we also observed an increased plasma AGT concentration in HFpEF mice models of two sexes (Figure 1L; Figure S1L, Supporting Information). These data revealed that HFpEF induced an elevation of AGT abundances in the liver and plasma.

### 2.2 Deficiency of Hepatic AGT Alleviated Diastolic Dysfunction in HFpEF Mice

**Figure 2:**
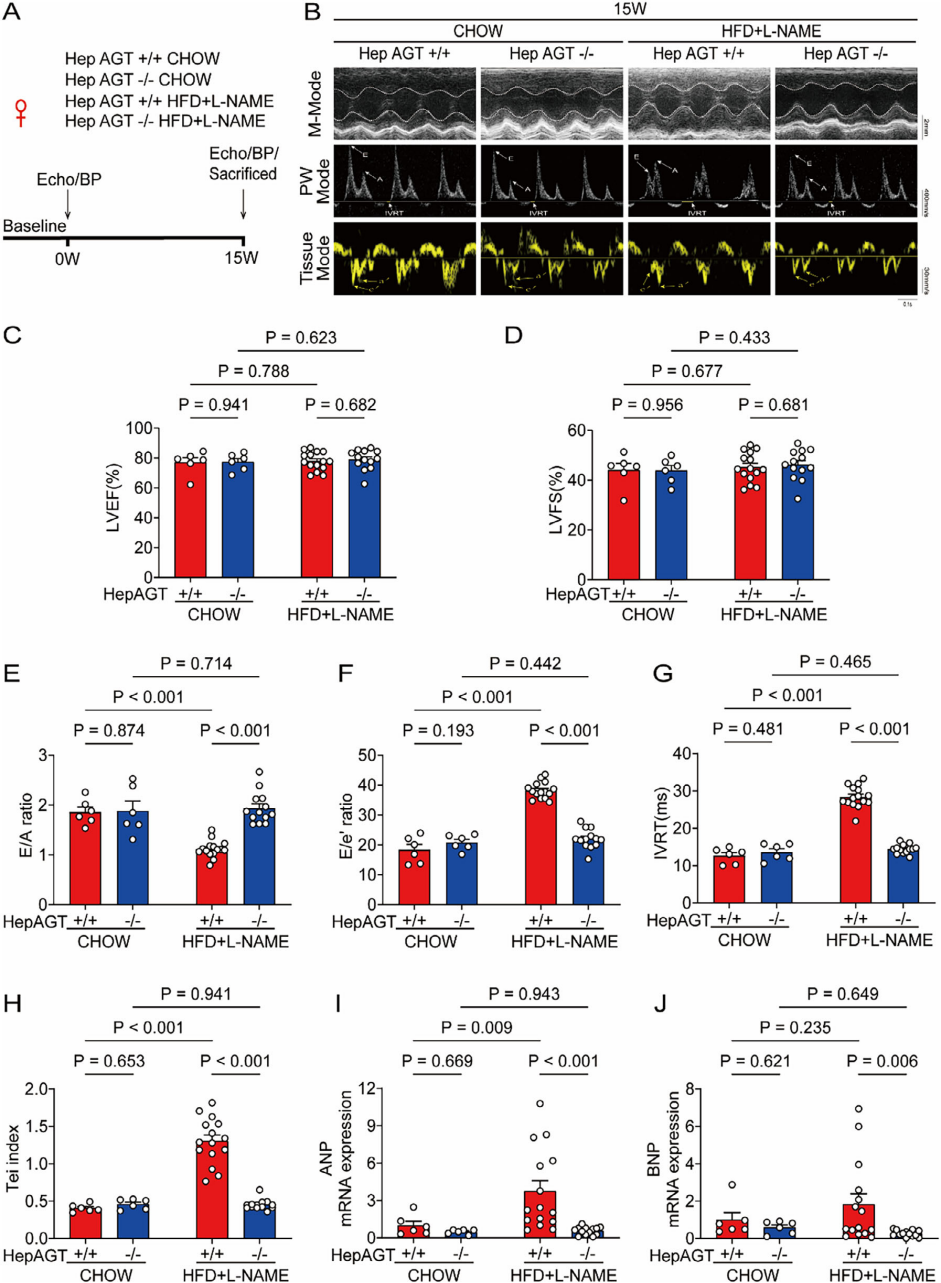
Deficiency of hepatic AGT alleviated diastolic dysfunction in female HFpEF mice. A) Experimental workflow and analysis for the effects of hepatic AGT deletion on HFpEF in female mice (n = 6 for hepAGT+/+ CHOW group, n = 6 for hepAGT‐/‐ CHOW group, n = 15 for hepAGT+/+ HFD+L‐NAME group, n = 13 for hepAGT‐/‐ HFD+L‐NAME group). B) Representative echocardiography images obtained from female mice at 15w after feeding with chow or HFD+L‐NAME (n = 6 for hepAGT+/+ CHOW group, n = 6 for hepAGT‐/‐ CHOW group, n = 15 for hepAGT+/+ HFD+L‐NAME group, n = 13 for hepAGT‐/‐ HFD+L‐NAME group). C) Left ventricular ejection fraction (LVEF%) was quantified via echocardiography (n = 6 for hepAGT+/+ CHOW group, n = 6 for hepAGT‐/‐ CHOW group, n = 15 for hepAGT+/+ HFD+L‐NAME group, n = 13 for hepAGT‐/‐ HFD+L‐NAME group). D) Left ventricular fraction shortening (LVFS%) was quantified via echocardiography (n = 6 for hepAGT+/+ CHOW group, n = 6 for hepAGT‐/‐ CHOW group, n = 15 for hepAGT+/+ HFD+L‐NAME group, n = 13 for hepAGT‐/‐ HFD+L‐NAME group). E) E/A ratio was quantified via echocardiography (n = 6 for hepAGT+/+ CHOW group, n = 6 for hepAGT‐/‐ CHOW group, n = 15 for hepAGT+/+ HFD+L‐NAME group, n = 13 for hepAGT‐/‐ HFD+L‐NAME group). F) E/e’ ratio was quantified via echocardiography (n = 6 for hepAGT+/+ CHOW group, n = 6 for hepAGT‐/‐ CHOW group, n = 15 for hepAGT+/+ HFD+L‐NAME group, n = 13 for hepAGT‐/‐ HFD+L‐NAME group). G) IVRT was quantified via echocardiography (n = 6 for hepAGT+/+ CHOW group, n = 6 for hepAGT‐/‐ CHOW group, n = 15 for hepAGT+/+ HFD+L‐NAME group, n = 13 for hepAGT‐/‐ HFD+L‐NAME group). H) Tei index was quantified via echocardiography (n = 6 for hepAGT+/+ CHOW group, n = 6 for hepAGT‐/‐ CHOW group, n = 15 for hepAGT+/+ HFD+L‐NAME group, n = 13 for hepAGT‐/‐ HFD+L‐NAME group). I) mRNA abundance of cardiac atrial natriuretic peptide (ANP) was assessed in female HFpEF hepAGT+/+ and female HFpEF hepAGT‐/‐ mice (n = 6 for hepAGT+/+ CHOW group, n = 6 for hepAGT‐/‐ CHOW group, n = 15 for hepAGT+/+ HFD+L‐NAME group, n = 13 for hepAGT‐/‐ HFD+L‐NAME group). J) mRNA abundance of cardiac brain natriuretic peptide (BNP) was assessed in female HFpEF hepAGT+/+ and female HFpEF hepAGT‐/‐ mice (n = 6 for hepAGT+/+ CHOW group, n = 6 for hepAGT‐/‐ CHOW group, n = 15 for hepAGT+/+ HFD+L‐NAME group, n = 13 for hepAGT‐/‐ HFD+L‐NAME group). Two‐way ANOVA was used for statistical analysis.

Since the liver is the primary source of plasma AGT, hepatocyte AGT specific knockout (hepAGT‐/‐) mice were used to determine whether hepAGT was responsible for HFpEF. The efficacy of hepAGT depletion was confirmed by Western blotting of liver tissues and measurement of plasma AGT concentrations (Figure S2A–D, Supporting Information). Interestingly, remarkable improvements in myocardial diastolic function were observed in female HFpEF hepAGT‐/‐ mice compared to female HFpEF hepAGT+/+ mice, as shown by increased E/A ratio, decreased E/e’ ratio, IVRT and left ventricular Tei index (**Figure** **2**A,B,E–H), although myocardial systolic function were similar between genotypes (Figure 2C,D). Furthermore, cardiac atrial natriuretic peptide (ANP) mRNA abundances were increased after HFpEF establishment (Figure 2I). Compared to HFpEF hepAGT+/+ littermates, cardiac ANP and brain natriuretic peptide (BNP) mRNA abundances were lower in HFpEF hepAGT‐/‐ mice (Figure 2I–J). Consistently, the above‐mentioned cardiac phenotypes were also reproduced in male HFpEF mice models (Figure S3A–J, Supporting Information).

Collectively, these observations supported that genetic ablation of AGT in hepatocytes ameliorated myocardial diastolic dysfunction in HFpEF mice.

### 2.3 Systemic Angiotensin II (AngII) Blockade Exhibited no Effects on Myocardial Diastolic Dysfunction in HFpEF Mice

**Figure 3:**
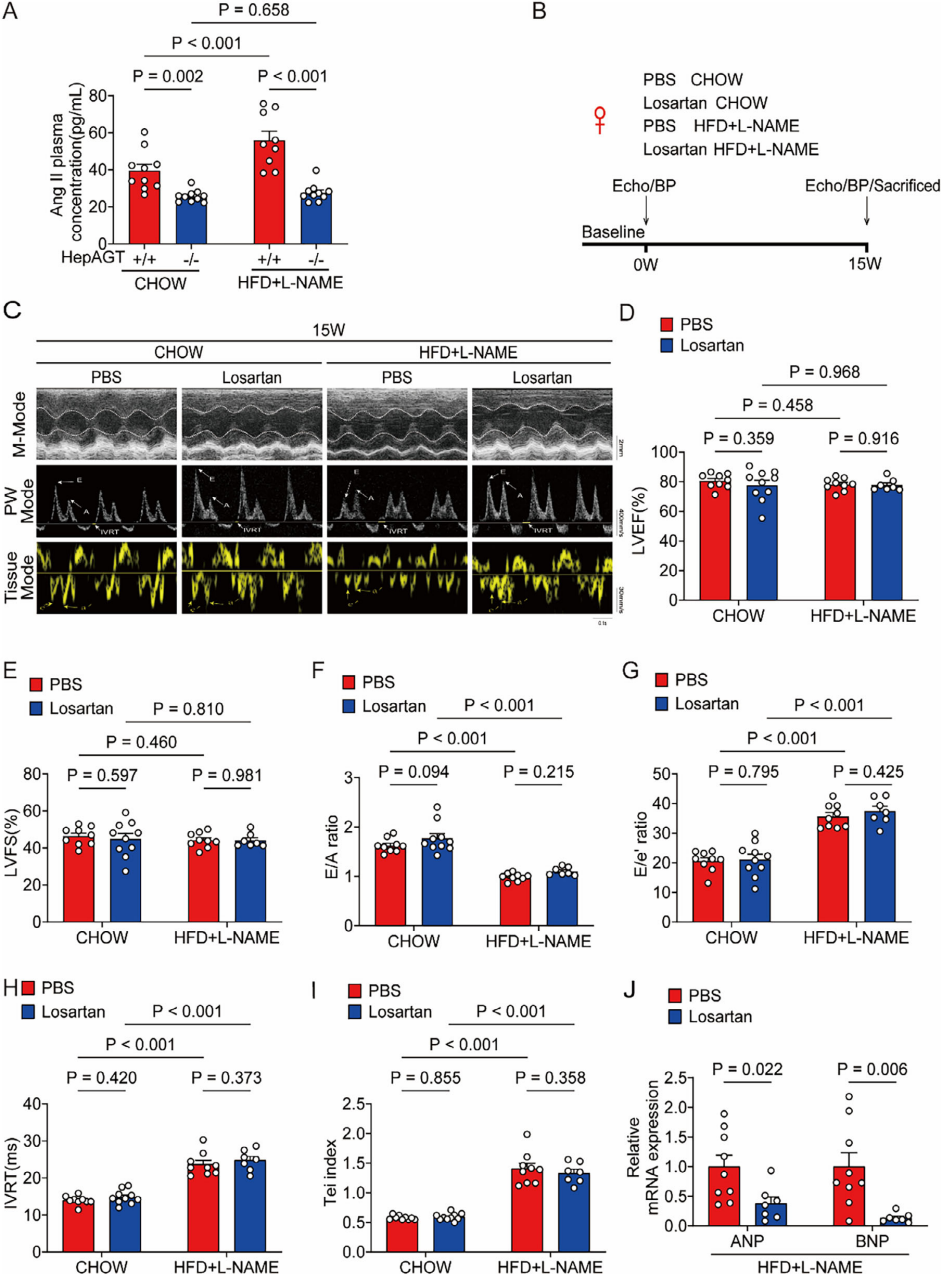
Inhibition of systemic AngII by losartan exhibited no effects on cardiac diastolic function in female HFpEF mice. A) Plasma AngII concentrations in female HFpEF hepAGT+/+ and female HFpEF hepAGT‐/‐ mice were measured by ELISA (n = 10 for hepAGT+/+ CHOW group, n = 10 for hepAGT‐/‐ CHOW group, n = 9 for hepAGT+/+ HFD+L‐NAME group, n = 10 for hepAGT‐/‐ HFD+L‐NAME group). B) Experimental workflow and analysis for the effects of losartan treatment on HFpEF in female mice (n = 9 for PBS CHOW group, n = 10 for Losartan CHOW group, n = 9 for PBS HFD+L‐NAME group, n = 7 for Losartan HFD+L‐NAME group). C) Representative echocardiography images obtained from female HFpEF mice at 15w after feeding with PBS or losartan (n = 9 for PBS CHOW group, n = 10 for Losartan CHOW group, n = 9 for PBS HFD+L‐NAME group, n = 7 for Losartan HFD+L‐NAME group). D) Left ventricular ejection fraction (LVEF%) was quantified via echocardiography (n = 9 for PBS CHOW group, n = 10 for Losartan CHOW group, n = 9 for PBS HFD+L‐NAME group, n = 7 for Losartan HFD+L‐NAME group). E) Left ventricular fraction shortening (LVFS%) was quantified via echocardiography (n = 9 for PBS CHOW group, n = 10 for Losartan CHOW group, n = 9 for PBS HFD+L‐NAME group, n = 7 for Losartan HFD+L‐NAME group). F) E/A ratio was quantified via echocardiography (n = 9 for PBS CHOW group, n = 10 for Losartan CHOW group, n = 9 for PBS HFD+L‐NAME group, n = 7 for Losartan HFD+L‐NAME group). G) E/e’ ratio was quantified via echocardiography (n = 9 for PBS CHOW group, n = 10 for Losartan CHOW group, n = 9 for PBS HFD+L‐NAME group, n = 7 for Losartan HFD+L‐NAME group). H) IVRT was quantified via echocardiography (n = 9 for PBS CHOW group, n = 10 for Losartan CHOW group, n = 9 for PBS HFD+L‐NAME group, n = 7 for Losartan HFD+L‐NAME group). I) Tei index was quantified via echocardiography (n = 9 for PBS CHOW group, n = 10 for Losartan CHOW group, n = 9 for PBS HFD+L‐NAME group, n = 7 for Losartan HFD+L‐NAME group). J) mRNA abundances of cardiac atrial natriuretic peptide (ANP) and cardiac brain natriuretic peptide (BNP) were assessed in female HFpEF mice feeding with PBS or losartan (n = 9 for PBS HFD+L‐NAME group, n = 7 for Losartan HFD+L‐NAME group). Two‐ way ANOVA was used for statistical analysis in A and D–I, and Student's *t* test was used for statistical analysis in J.

AGT is cleaved by renin to produce angiotensin I (AngI). Subsequently, angiotensin‐converting enzyme (ACE) catalyzes the conversion of AngI into AngII, which functions as the pivotal effector peptide of the RAS.^[^ ^29^ ^]^ Therefore, plasma AngII concentrations from HFpEF hepAGT‐/‐ mice and their HFpEF hepAGT+/+ littermates were determined both in females and males. Plasma AngII concentrations were slightly increased after HFpEF establishment, and lower in HFpEF hepAGT‐/‐ mice (**Figure** **3**A; Figure S5A, Supporting Information), indicating that hepatic AGT deletion could suppress RAS after HFpEF induction.

To further investigate whether an AngII‐dependent pathway contributes to HFpEF, an angiotensin receptor type 1 (AT1R) antagonist, losartan, was used to verify whether blocking AngII‐AT1R signaling simulated cardiac phenotypes of hepAGT‐/‐ mice in the HFpEF model. Losartan treatment could decrease blood pressure both in female and male HFpEF hepAGT+/+ mice (Figure S4A–D, Supporting Information). However, HFpEF hepAGT+/+ mice treated with losartan displayed similar myocardial diastolic dysfunction compared to those treated with PBS, both in female and male (Figure 3B–I; Figure S5B–I, Supporting Information). Of note, cardiac ANP and BNP mRNA abundances were all decreased in losartan‐treated HFpEF hepAGT+/+ mice versus PBS‐treated HFpEF hepAGT+/+ mice (Figure 3J; Figure S5J, Supporting Information). These findings implicated that systemic inhibition of AngII failed to improve cardiac diastolic function in HFpEF mouse models, which aligns with current clinical research outcomes, suggesting that therapeutic strategies targeting AngII‐dependent pathways may inadequately address the core pathological mechanisms underlying HFpEF.

The pathogenesis of HFpEF is highly heterogeneous, involving multifactorial pathological mechanisms including metabolic dysregulation, inflammation, myocardial fibrosis, ventricular hypertrophy, and microvascular dysfunction.^[^ ^30^, ^31^, ^32^, ^33^, ^34^, ^35^, ^36^, ^37^, ^38^, ^39^ ^]^ To address this complexity, the present study investigates the regulatory role of hepatic AGT in HFpEF through the above‐mentioned pathological axes:

### 2.4 Sex Differences in Metabolic Phenotypes regulated by hepAGT in HFpEF Mice Model

Since patients with HFpEF are usually accompanied by metabolic syndrome, including hypertension, hyperlipidemia, obesity, and insulin resistance,^[^ ^31^, ^32^, ^33^ ^]^ we then measured blood pressures, lipid profiles, body weight as well as blood glucose of hepAGT+/+ and hepAGT‐/‐ mice. In both female and male hepAGT+/+ mice, systolic and diastolic blood pressures were all increased after HFpEF induction. HFpEF hepAGT‐/‐ mice had lower systolic and diastolic blood pressures compared to HFpEF hepAGT+/+ mice (Figure S6A,E, Supporting Information). In addition, after HFpEF was induced, plasma total cholesterol (TC) and total triglycerides (TG) concentrations showed no statistical difference between genotypes (Figure S6B,F, Supporting Information).

Body weight was monitored continuously after feeding with HFD+L‐NAME. In female mice, after 5 weeks of HFD+L‐NAME feeding, hepAGT‐/‐ mice started to exhibit significantly less body weight gain than hepAGT+/+ mice. At the end of the 15‐week‐HFD+L‐NAME feeding, the body weight gain of hepAGT‐/‐ mice was significantly lower than that of hepAGT+/+ counterparts (Figure S6C, Supporting Information). However, body weight gain showed no statistical difference between genotypes in male mice (Figure S6G, Supporting Information).

Additionally, in cohorts of female mice fed with HFD+L‐NAME, the incremental area under the curve of the blood glucose was reduced in the hepAGT‐/‐ group compared with the hepAGT+/+ group, which implicated that glucose tolerance was improved in female hepAGT‐/‐ mice after HFpEF induction (Figure S6D, Supporting Information). Unexpectedly, these phenotypes could not be reproduced in male mice (Figure S6H, Supporting Information).

Collectively, all these data revealed a sex discrepancy in body weight gain and glucose metabolism regulated by hepAGT in the HFpEF model, indicating that metabolic modulation may not be a shared mechanism for hepatic AGT to regulate HFpEF in mice of two sexes.

### 2.5 HepAGT Depletion Exhibited No Effects on Cardiac Fibrosis and Inflammation in HFpEF Mice

Other than metabolic stress, cardiac fibrosis and inflammation are the two major mechanisms contributing to the development of HFpEF.^[^ ^34^, ^36^ ^]^ We then investigated the effects of hepAGT deficiency on cardiac fibrosis and inflammation. In both female and male mice, cardiac fibrotic area (Figure S7A,I, Supporting Information) as well as the abundances of myocardial fibrotic markers including Col1α1, Col1α2, Col3α1, Postn, Fn1, ACTA2 and TGF‐β displayed no statistical differences between genotypes in HFpEF mice of either sex (Figure S7B–H,J–P, Supporting Information). In addition, we did not observe significant differences in cardiac inflammatory genes expression including IL‐1β, IL‐6 as well as TNF‐α between genotypes in both female and male HFpEF mice (Figure S9A–F, Supporting Information).

The abundances of these fibrotic markers and inflammatory genes were also systematically evaluated in HFpEF mouse models treated with losartan. The results revealed that losartan treatment failed to significantly alter cardiac fibrotic or inflammatory responses in HFpEF mice of either sex (Figures S8A–N, S10A–F, Supporting Information).

Collectively, these data suggest that cardiac fibrosis and inflammation are not the key contributors to hepatic AGT‐regulated HFpEF in male and female mice.

### 2.6 Deficiency of Hepatic AGT Attenuated Myocardial Hypertrophy in HFpEF Mice

Given that the RAS has a profound impact on myocardial hypertrophy and myocardial hypertrophy contributes to the development of HFpEF,^[^ ^40^ ^]^ heart weight and cardiomyocytes surface area of hepAGT+/+ and hepAGT‐/‐ mice were then measured. After induction of HFpEF, heart weight and cardiomyocytes surface area were significantly decreased in hepAGT‐/‐ mice compared to hepAGT+/+ littermates, both in male and female (Figure S11A–D, Supporting Information).

In addition, we observed that losartan treatment could remarkably decrease heart weight as well as reduce cardiomyocytes surface area (Figure S11E–H, Supporting Information) in HFpEF mice of either sex. All these data suggested that hepAGT deficiency could attenuate myocardial hypertrophy after HFpEF induction, which was AngII‐dependent.

### 2.7 Deficiency of Hepatic AGT Promoted Microvascular Angiogenesis in HFpEF Mice

**Figure 4:**
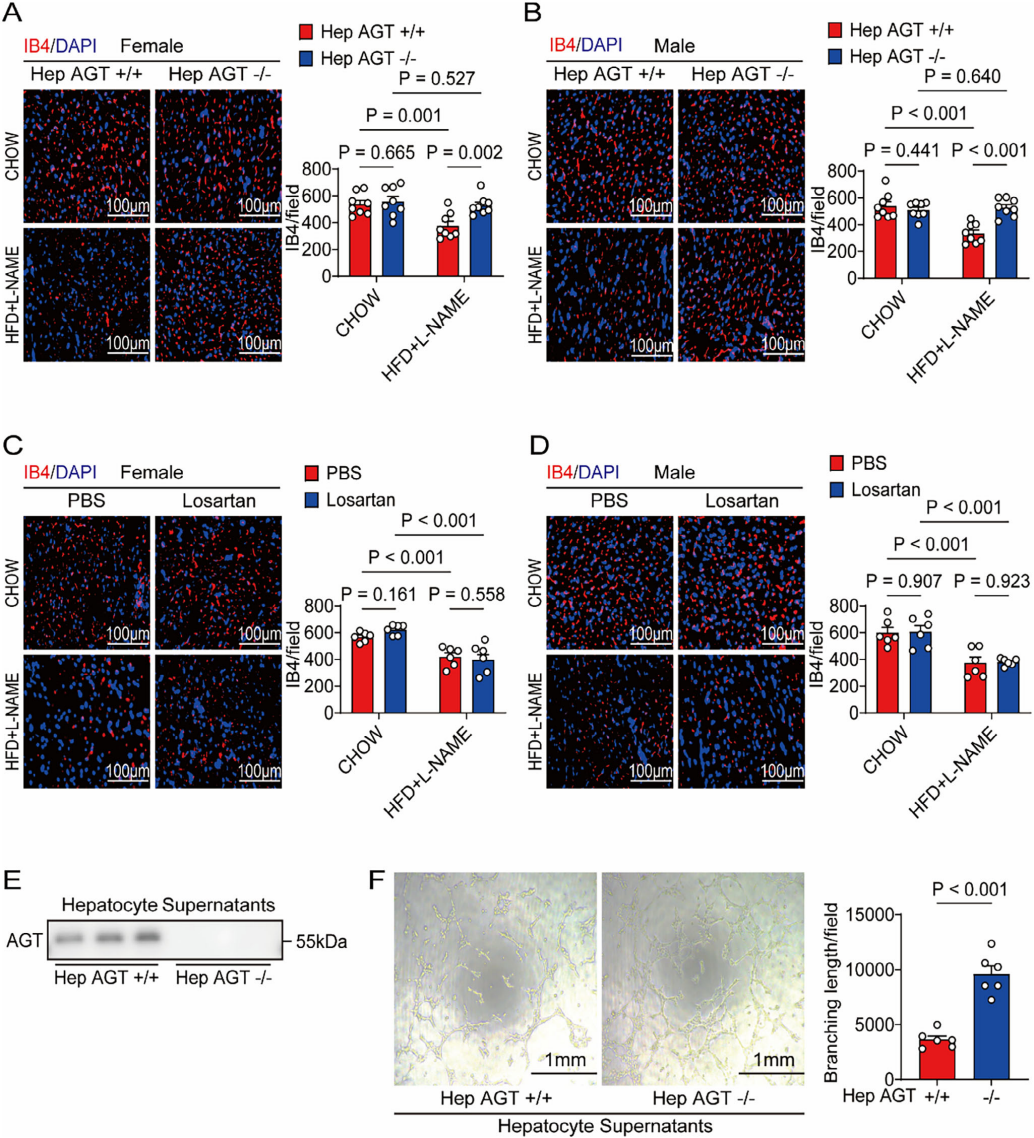
Deficiency of hepatic AGT promoted microvascular angiogenesis in HFpEF mice. A) Representative IB4 immune‐staining images of cardiac tissues obtained from female hepAGT+/+ and female hepAGT‐/‐ mice at 15w after feeding with Chow or HFD+L‐NAME, then IB4 density of cardiac tissues was calculated in female HFpEF hepAGT+/+ and female HFpEF hepAGT‐/‐ mice (n = 8 for each group, Bar = 100 µm). B) Representative IB4 immune‐staining images of cardiac tissues obtained from male hepAGT+/+ and male hepAGT‐/‐ mice at 15w after feeding with Chow or HFD+L‐NAME, then IB4 density of cardiac tissues was calculated in male HFpEF hepAGT+/+ and male HFpEF hepAGT‐/‐ mice (n = 8 for each group, Bar = 100 µm). C) Representative IB4 immune‐staining images of cardiac tissues obtained from female HFpEF mice at 15w after feeding with PBS or losartan, then IB4 density of cardiac tissues was calculated in female HFpEF mice fed with PBS or losartan (n = 6 for each group, Bar = 100 µm). D) Representative IB4 immune‐staining images of cardiac tissues obtained from male HFpEF mice at 15w after feeding with PBS or losartan, then IB4 density of cardiac tissues was calculated in male HFpEF mice fed with PBS or losartan (n = 6 for each group, Bar = 100 µm). E) Western blotting detection of AGT protein abundances in hepatocyte supernatants obtained from HFpEF hepAGT+/+ and HFpEF hepAGT‐/‐ mice (n = 3 for each group). F) Mouse cardiac endothelial cell lines (MCECs) were incubated with hepatocyte supernatants obtained from HFpEF hepAGT+/+ and HFpEF hepAGT‐/‐ mice. Subsequently, the representative tube formation images were acquired (n = 6 for each group). Tube lengths were finally calculated in hepAGT+/+ and hepAGT‐/‐ groups, respectively (n = 6 for each group, Bar = 1mm). Two‐ way ANOVA was used for statistical analysis in A–D, and Student's t test was used for statistical analysis in F.

Previous studies have found that the capillary density of myocardial tissues in patients with HFpEF is significantly lower than that of normal controls. Further high‐throughput sequencing also confirmed that the expression of key genes of the angiogenesis signaling pathway in cardiac tissues was significantly down‐regulated in HFpEF patients, indicating that capillary loss is a vital mechanism contributing to HFpEF.^[^ ^35^, ^41^ ^]^ Therefore, we investigated the effect of hepatic AGT deficiency on cardiac angiogenesis in HFpEF mice models. In both female and male mice, HFD+L‐NAME treatment could induce microvascular rarefaction as displayed by reduction of IB4+ cells infiltration, while hepatic AGT deficiency could partially reverse these phenotypes (**Figure** **4**A,B). However, no significant differences in α‐SMA+ cells infiltration in heart tissues were observed between HFpEF hepAGT‐/‐ mice and HFpEF hepAGT+/+ mice (Figure S12A,B, Supporting Information). In vitro an enhanced tube formation was observed in mouse cardiac endothelial cells (MCECs) incubated with hepatocyte supernatants collected from HFpEF hepAGT‐/‐ mice (Figure 4E,F). Additionally, losartan treatment had no effects on myocardial microvascular angiogenesis in HFpEF mice of either sex (Figure 4C,D).

Collectively, we observed that hepatic AGT knockout significantly ameliorated cardiac diastolic dysfunction in HFpEF mice, whereas systemic AngII inhibition (losartan) failed to replicate this therapeutic effect. Further mechanistic screening revealed that both interventions (hepatic AGT knockout vs systemic AngII inhibition) showed similar outcomes in terms of cardiac inflammation, myocardial fibrosis, and ventricular hypertrophy. However, they exhibited divergent effects on myocardial microvascular density and angiogenic capacity. These findings suggested that hepatic AGT may regulate HFpEF pathogenesis through an AngII‐independent mechanism centered on microvascular angiogenesis, highlighting this pathway as a pivotal contributor to HFpEF progression.

### 2.8 Deficiency of Hepatic AGT Promoted Cardiac Microvascular Angiogenesis by Activating GATA2/Pim3 Pathway

We then attempted to define the mechanism through which hepatic AGT regulated cardiac microvascular angiogenesis in HFpEF. Because hepatic AGT exerts similar effects on microvascular angiogenesis in both HFpEF female and HFpEF male mice, we then chose female mice for the subsequent mechanistic study.

**Figure 5:**
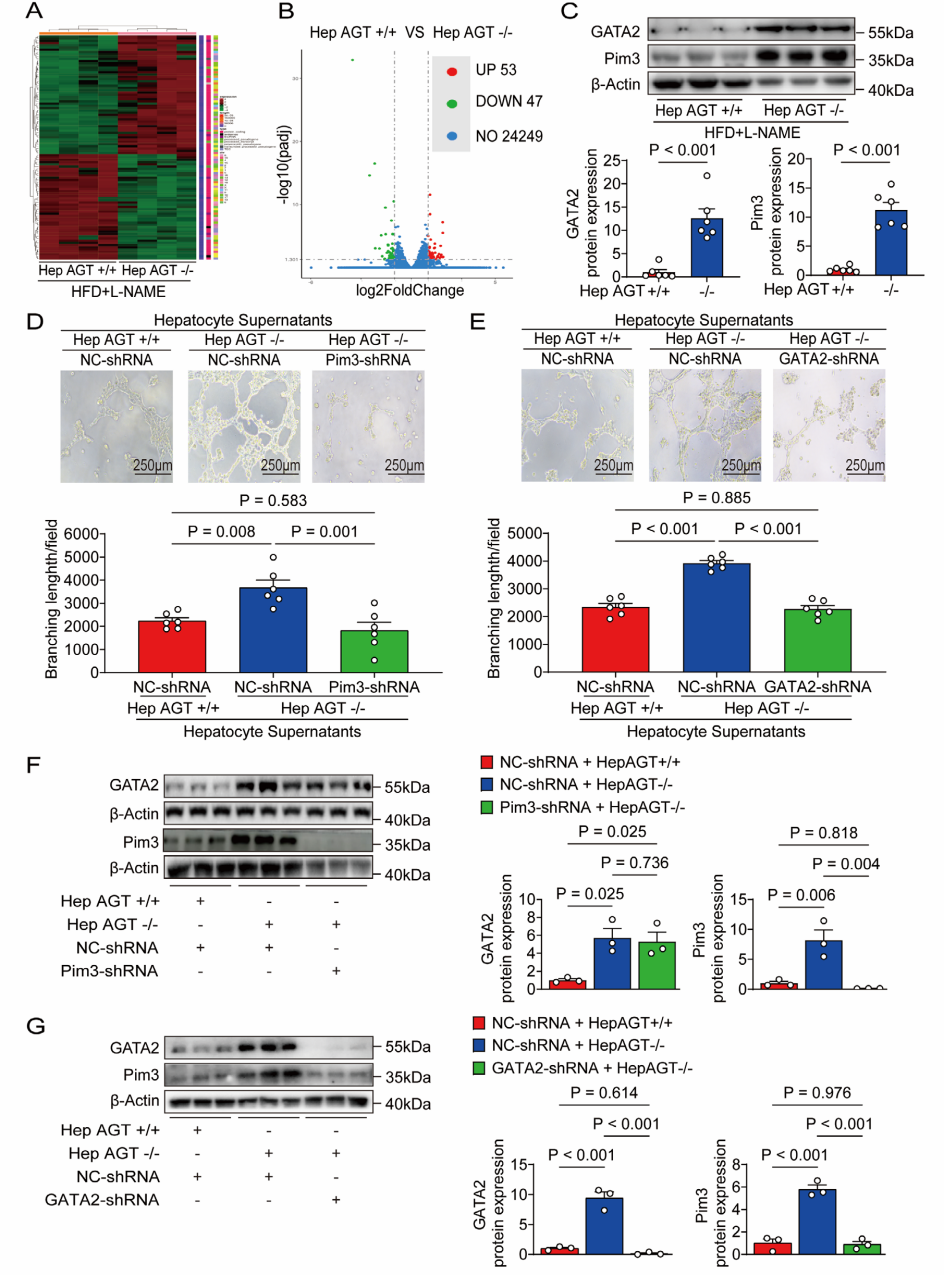
Deficiency of hepatic AGT promoted cardiac microvascular angiogenesis by activating the GATA2/Pim3 pathway. A) Clustered heat map of genes between hearts from HFpEF hepAGT‐/‐ mice and HFpEF hepAGT+/+ mice by RNA‐seq analysis (n = 4 for each group). B) Funnel plot images of genes between hearts from HFpEF hepAGT‐/‐ mice and HFpEF hepAGT+/+ mice by RNA‐seq analysis (n = 4 for each group). C) Western blotting detection and quantification of GATA2 and Pim3 protein abundances in hearts obtained from female HFpEF hepAGT+/+ and female HFpEF hepAGT‐/‐ mice (n = 6 for each group). D) Representative tube formation images and calculation of tube lengths were obtained from MCECs transfected with either scrambled or Pim3 shRNA incubated with hepatocyte supernatants derived from hepAGT+/+ and hepAGT‐/‐ mice (n = 6 for each group, Bar = 250µm). E) Representative tube formation images and calculation of tube lengths were obtained from MCECs transfected with either scrambled or GATA2 shRNA incubated with hepatocyte supernatants derived from hepAGT+/+ or hepAGT‐/‐ mice (n = 6 for each group, Bar = 250µm). F) Western blotting detection of GATA2 and Pim3 protein abundances in MCECs transfected with either scrambled or Pim3 shRNA incubated with hepatocyte supernatants derived from hepAGT+/+ or hepAGT‐/‐ mice (n = 3 for each group). G) Western blotting detection of GATA2 and Pim3 protein abundances in MCECs transfected with either scrambled or GATA2 shRNA incubated with hepatocyte supernatants derived from hepAGT+/+ or hepAGT‐/‐ mice (n = 3 for each group). Student's *t* test was used for statistical analysis in C. One‐ way ANOVA was used for statistical analysis in D–G.

RNA‐sequencing analysis was performed on heart tissues collected from hepAGT+/+ and hepAGT‐/‐ mice 15 weeks after HFD+L‐NAME treatment. A clustered heat map was generated between HFpEF hepAGT+/+ and HFpEF hepAGT‐/‐ mice (criterion: *P* < 0.05) (**Figure** **5**A). Among the 53 genes that were significantly upregulated, Pim‐3 Proto‐Oncogene, Serine/Threonine Kinase (Pim3) was significantly altered (Figure 5B). Consistent with transcriptome data, protein abundances of Pim3 were increased in HFpEF hepAGT‐/‐ hearts compared to HFpEF hepAGT+/+ counterparts from both female and male mice (Figure 5C; Figure S13A, Supporting Information). Consistently, in vitro an elevated Pim3 protein level was observed in MCECs incubated with supernatants collected from HFpEF hepAGT‐/‐ mouse hepatocytes (Figure S13B, Supporting Information). However, no significant alteration in cardiac Pim3 protein expression level was observed between HFpEF mice treated with losartan and those administered phosphate‐buffered saline (PBS), irrespective of sex (Figure S13C,D, Supporting Information), suggesting that hepAGT mediated the elevation of cardiac Pim3 abundances may be AngII‐independent. Pim3 is a member of the Moloney murine leukemia virus (Pim) family and belongs to the Ca2+/calmodulin‐dependent protein kinase (CaMK) with serine/threonine activity. Pim3 is widely expressed in various tumor cells and endothelial cells, exerting pro‐angiogenic effects, and ultimately promoting the development of tumors.^[^ ^42^, ^43^ ^]^ Based on these findings, we hypothesized that hepatic AGT mediated Pim3 expression in cardiac endothelial cells may be one of the key pathways to regulate HFpEF.

To verify this hypothesis, the tube length of Pim3‐depleted or control MCECs incubated with HFpEF hepAGT+/+ or HFpEF hepAGT‐/‐ mouse hepatocyte‐derived supernatants, respectively, was examined. The tube length in HFpEF hepAGT‐/‐ mouse hepatocytes supernatants‐incubated control MCECs increased significantly compared to HFpEF hepAGT+/+ mouse hepatocytes supernatants‐incubated control MCECs. However, tube formation was suppressed substantially in Pim3‐depleted MCECs incubated with HFpEF hepAGT‐/‐ mouse hepatocytes supernatants (Figure 5D). Collectively, these observations demonstrated that a deficiency of hepatic AGT promoted cardiac microvascular angiogenesis in HFpEF by increasing Pim3 abundances.

Previous studies have shown that the expression of Pim3 is regulated by transcription factors.^[^ ^44^ ^]^ To gain insight into which transcription factor was responsible for hepAGT mediated cardiac Pim3 modulation, we reviewed the RNA‐sequencing data, analyzed the expression patterns of endothelial‐specific transcription factors, and found enhanced expression of GATA Binding Protein 2 (GATA2) in HFpEF hepAGT‐/‐ mice hearts (Figure 5B). Next immune blotting revealed that protein abundances of GATA2 were increased in HFpEF hepAGT‐/‐ hearts compared to HFpEF hepAGT+/+ counterparts (Figure 5C; Figure S13A, Supporting Information), while no significant alteration in cardiac GATA2 protein expression level was observed between HFpEF mice treated with losartan and those administered PBS (Figure S13C,D, Supporting Information). In vitro an enhanced GATA2 protein abundance was observed in MCECs incubated with supernatants collected from HFpEF hepAGT‐/‐ mouse hepatocytes (Figure S13B, Supporting Information). Then control MCECs and GATA2 depleted MCECs were incubated with HFpEF hepAGT‐/‐ hepatocyte‐derived supernatants, respectively, and tube formation was substantially suppressed after deficiency of GATA2 (Figure 5E). Finally, we investigated whether GATA2 could modulate Pim3 expression in MCECs. No significant difference in GATA2 protein abundances was observed between Pim3 depleted MCECs and control MCECs incubated with HFpEF hepAGT‐/‐ hepatocyte‐derived supernatants, respectively (Figure 5F). However, the expression of Pim3 in MCECs was substantially suppressed after deficiency of GATA2 (Figure 5G), indicating that Pim3 is the downstream target of GATA2. To determine whether GATA2 could bind to the Pim3 gene promoter, a dual‐luciferase assay was further performed, and demonstrated that GATA2 plasmids significantly promoted the Pim3 transcriptional activity in HEK293T cells. This effect could be hindered by mutation of the Pim3‐binding motif (Figure S13E, Supporting Information). Collectively, these observations revealed a GATA2/Pim3 pathway contributing to cardiac microvascular angiogenesis regulated by hepAGT in HFpEF, which was AngII‐independent.

### 2.9 LRP2 was required for Hepatocyte‐Derived AGT Internalization in Cardiac Endothelial Cells

**Figure 6:**
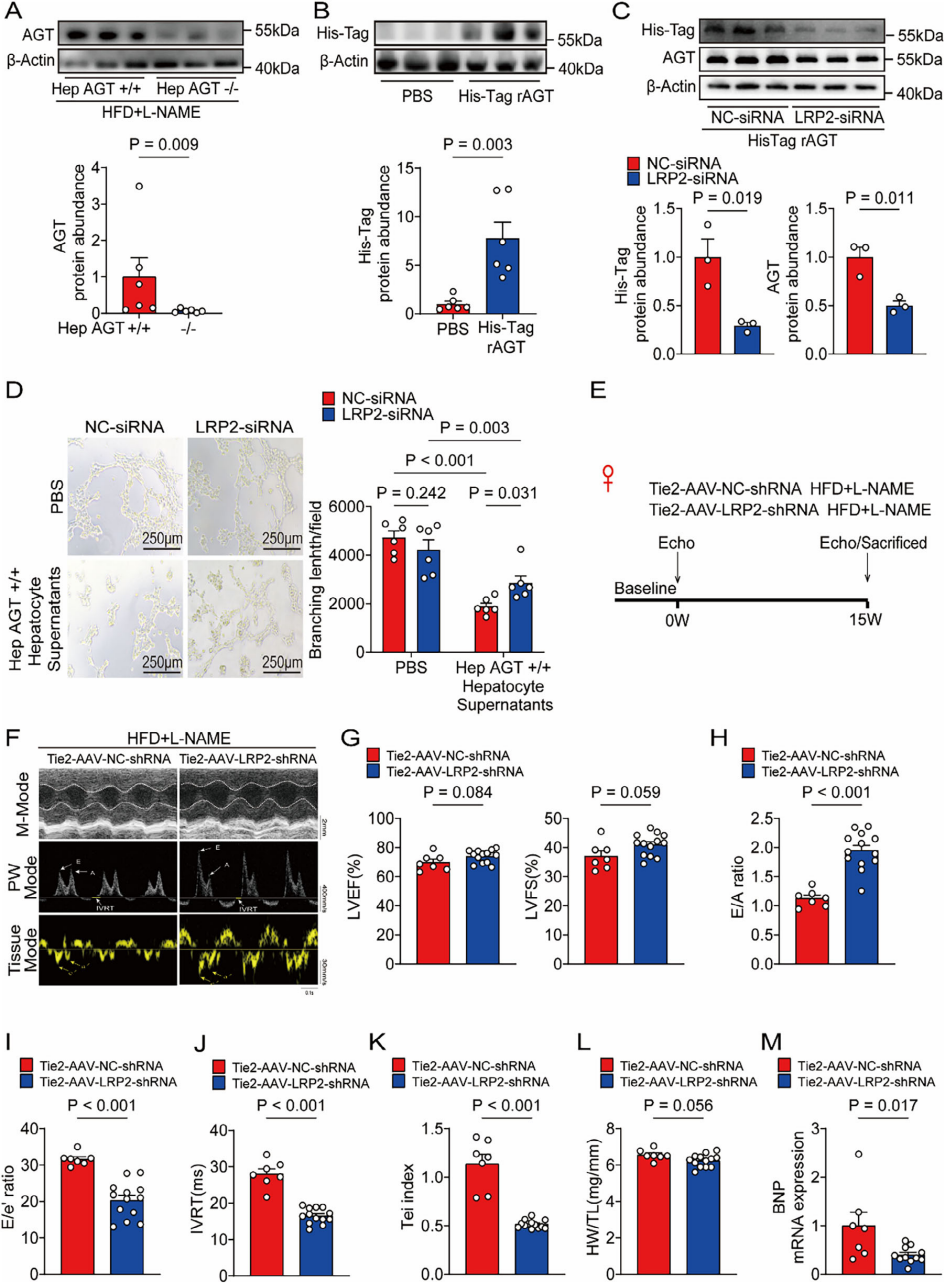
LRP2 was required for hepatocyte‐derived AGT internalization in cardiac endothelial cells. A) Western blotting detection and quantification of AGT protein abundances in hearts obtained from female HFpEF hepAGT+/+ and female HFpEF hepAGT‐/‐ mice (n = 6 for each group). B) MCECs were incubated with His‐Tag labelled rAGT protein or PBS, and the His‐Tag protein abundances of MCECs were analyzed by Western blotting (n = 6 for each group). C) MCECs transfected with either scrambled or LRP2 siRNA were incubated with His‐Tag labelled rAGT protein or PBS, and the His‐Tag and AGT protein abundances of MCECs were analyzed by Western blotting (n = 3 for each group). D) Representative tube formation images and calculation of tube lengths obtained from MCECs transfected with either scrambled or LRP2 siRNA incubated with hepatocyte supernatants derived from hepAGT+/+ mice (n = 6 for each group, Bar = 250µm). E) Experimental workflow and analysis for the effects of Tie2‐AAV‐LRP2‐shRNA myocardial injection on HFpEF in female mice (n = 7 for Tie2‐AAV‐NC‐shRNA, and n = 13 for Tie2‐AAV‐LRP2‐shRNA). F) Representative echocardiography images obtained from female HFpEF mice at 15w after injection with Tie2‐AAV‐LRP2‐shRNA (n = 7 for Tie2‐AAV‐NC‐shRNA, and n = 13 for Tie2‐AAV‐LRP2‐shRNA). G) Left ventricular ejection fraction (LVEF%) and left ventricular fraction shortening (LVFS%) were quantified via echocardiography (n = 7 for Tie2‐AAV‐NC‐shRNA, and n = 13 for Tie2‐AAV‐LRP2‐shRNA). H) E/A ratio was quantified via echocardiography (n = 7 for Tie2‐AAV‐NC‐shRNA, and n = 13 for Tie2‐AAV‐LRP2‐shRNA). I) E/e’ ratio was quantified via echocardiography (n = 7 for Tie2‐AAV‐NC‐shRNA, and n = 13 for Tie2‐AAV‐LRP2‐shRNA). J) IVRT was quantified via echocardiography (n = 7 for Tie2‐AAV‐NC‐shRNA, and n = 13 for Tie2‐AAV‐LRP2‐shRNA). K) Tei index was quantified via echocardiography (n = 7 for Tie2‐AAV‐NC‐shRNA, and n = 13 for Tie2‐AAV‐LRP2‐shRNA). L) Ratio of heart weight to tibia length (HW/TL) was measured in female HFpEF mice injected with Tie2‐AAV‐LRP2‐shRNA (n = 7 for Tie2‐AAV‐NC‐shRNA, and n = 13 for Tie2‐AAV‐LRP2‐shRNA). M) mRNA abundance of cardiac brain natriuretic peptide (BNP) was assessed in female HFpEF mice injected with Tie2‐AAV‐LRP2‐shRNA (n = 7 for Tie2‐AAV‐NC‐shRNA, and n = 11 for Tie2‐AAV‐LRP2‐shRNA). Student's *t* test was used for statistical analysis in B‐C and G‐M. Mann‐Whitney U test was used for statistical analysis in A. Two‐way ANOVA was used for statistical analysis in D.

We next identify how the liver synthesizes AGT to remotely regulate cardiac angiogenic signals, which ultimately affects the development of HFpEF. Our previous investigation demonstrated that hepatic AGT deletion in septic myocardial dysfunction models significantly reduced cardiac AGT expression and ameliorated myocardial inflammation. Mechanistic studies revealed that cardiac fibroblasts internalize circulating liver‐derived AGT, thereby activating the NLRP3 inflammasome pathway and exacerbating cardiac dysfunction.^[^ ^45^ ^]^ Intriguingly, in our current HFpEF model studies, hepatic AGT knockout similarly downregulated cardiac AGT expression (**Figure** **6**A), concomitant with markedly enhanced myocardial microvascular angiogenesis. Building upon these findings, we hypothesized that cardiac endothelial cells may internalize hepatic AGT, which potentially suppresses angiogenesis. This mechanism might crucially contribute to the pathogenesis of HFpEF.

To validate our hypothesis, cell lysates from MCECs incubated with His‐Tag labelled recombinant AGT (rAGT) protein or PBS were examined. The amount of internalized rAGT in MCECs increased significantly compared to the PBS‐incubated group (Figure 6B), indicating that exogenous AGT could be accumulated in MCECs. We next investigated how MCECs accumulated hepAGT. Current evidences from both published literatures and our previous investigations have demonstrated that the LDL receptor‐related protein 1 (LRP1) and LDL receptor‐related protein 2 (LRP2) represent the exclusive receptors mediating the uptake of circulation AGT, therefore, qPCR was performed to explore the transcriptional abundances of LRP1 and LRP2, and found that only LRP2 was expressed predominantly in MCECs (Figure S14A, Supporting Information). Immunofluorescence staining revealed that LRP2, not LRP1, was highly expressed in MCECs (Figure S14B, Supporting Information). Additionally, the enhanced abundances of LRP2 mRNA were also detected in endothelial cells isolated from cardiac tissues (Figure S14C, Supporting Information), suggesting that LRP2 was highly expressed specifically in cardiac endothelial cells. Cell lysates from LRP2‐depleted or control MCECs incubated with His‐Tag labelled rAGT protein were then examined, and found that internalization was suppressed substantially in LRP2‐depleted MCECs (Figure 6C; Figure S14D,E, Supporting Information). The tube length in LRP2‐depleted MCECs increased significantly compared to control MCECs after incubation with HFpEF hepAGT+/+ mouse hepatocytes supernatants, respectively (Figure 6D).

To further verify these results in vivo, an adeno‐associated‐virus (AAV) with endothelial specific promoter Tie2 encoding short hairpin RNA (shRNA) targeting LRP2 (Tie2‐AAV‐LRP2‐shRNA) was generated, and the scrambled (NC) Tie2‐AAV (Tie2‐AAV‐NC‐shRNA) was constructed as controls. AAVs were then injected into the myocardium at the beginning of HFpEF model induction (Figure 6E). The efficacy of in vivo LRP2 reduction was confirmed by qPCR of cardiac endothelial cells sorted from whole heart tissues administered Tie2‐AAV‐LRP2‐shRNA (Figure S14F, Supporting Information). Interestingly, the AGT protein levels in heart tissues were decreased profoundly after Tie2‐AAV‐LRP2‐shRNA injection, indicating that exogenous AGT may be internalized into cardiac endothelial cells via LRP2 (Figure S14G, Supporting Information). As expected, the improved cardiac diastolic function (Figure 6F–K), a trend toward decreased heart weight (Figure 6L), enhanced cardiac microvascular angiogenesis (Figure S14H, Supporting Information), reduction of cardiac BNP mRNA abundances (Figure 6M) as well as increased expression of cardiac GATA2 and Pim3 (Figure S14I, Supporting Information) were observed in female HFpEF mice administrated Tie2‐AAV‐LRP2‐shRNA. In summary, these results revealed an LRP2‐dependent mechanism contributing to hepAGT internalization in cardiac endothelial cells, subsequently inhibiting cardiac microvascular angiogenesis, ultimately promoting HFpEF development.

### 2.10 Calcium‐Handling Dysregulation May Mediate the Connection between hepAGT‐Driven Microvascular Rarefaction and Diastolic Dysfunction

Hepatic AGT deficiency alleviated diastolic dysfunction, likely by attenuating microvascular rarefaction‐induced myocardial hypoperfusion. Although myocardial hypoperfusion may impair diastolic function through mechanisms including inflammation, fibrosis, calcium dysregulation, impaired energetics, or reduced diastolic perfusion,^[^ ^35^, ^46^, ^47^, ^48^ ^]^ neither cardiac fibrosis nor inflammation changed in hepatic AGT‐regulated HFpEF pathogenesis. Given our previous finding that hepAGT impairs cardiac function via calcium dysregulation in acute cardiac injury models, we focused on calcium‐handling pathways. Critically, phospholamban (PLN) phosphorylation status regulates SERCA2a activity. Enhanced PLN phosphorylation accelerates sarcoplasmic reticulum calcium reuptake via SERCA2a activation, improving diastolic relaxation.^[^ ^49^, ^50^, ^51^, ^52^ ^]^ We therefore analyzed key calcium‐handling proteins in HFpEF hepAGT‐/‐ models. Cardiac tissue analysis revealed significantly increased PLN phosphorylation (p‐PLN/PLN ratio) in HFpEF hepAGT‐/‐ mice without altered SERCA2a or titin expression (Figure S15A–D, Supporting Information). Furthermore, specific knockdown of LRP2 in cardiac endothelial cells to block hepatic‐derived AGT entry heart, followed by HFpEF induction, similarly elevated cardiac PLN phosphorylation levels without significantly altering SERCA2a or titin expression (Figure S15E–H, Supporting Information). These findings indicate that enhanced PLN phosphorylation improves calcium handling, thereby likely mediating the ameliorated diastolic function resulting from hepatic AGT deficiency‐induced attenuation of microvascular rarefaction.

### 2.11 18β‐glycyrrhetinic Acid (18β‐GA) is a Potent Suppressor of hepAGT to Treat HFpEF

**Figure 7:**
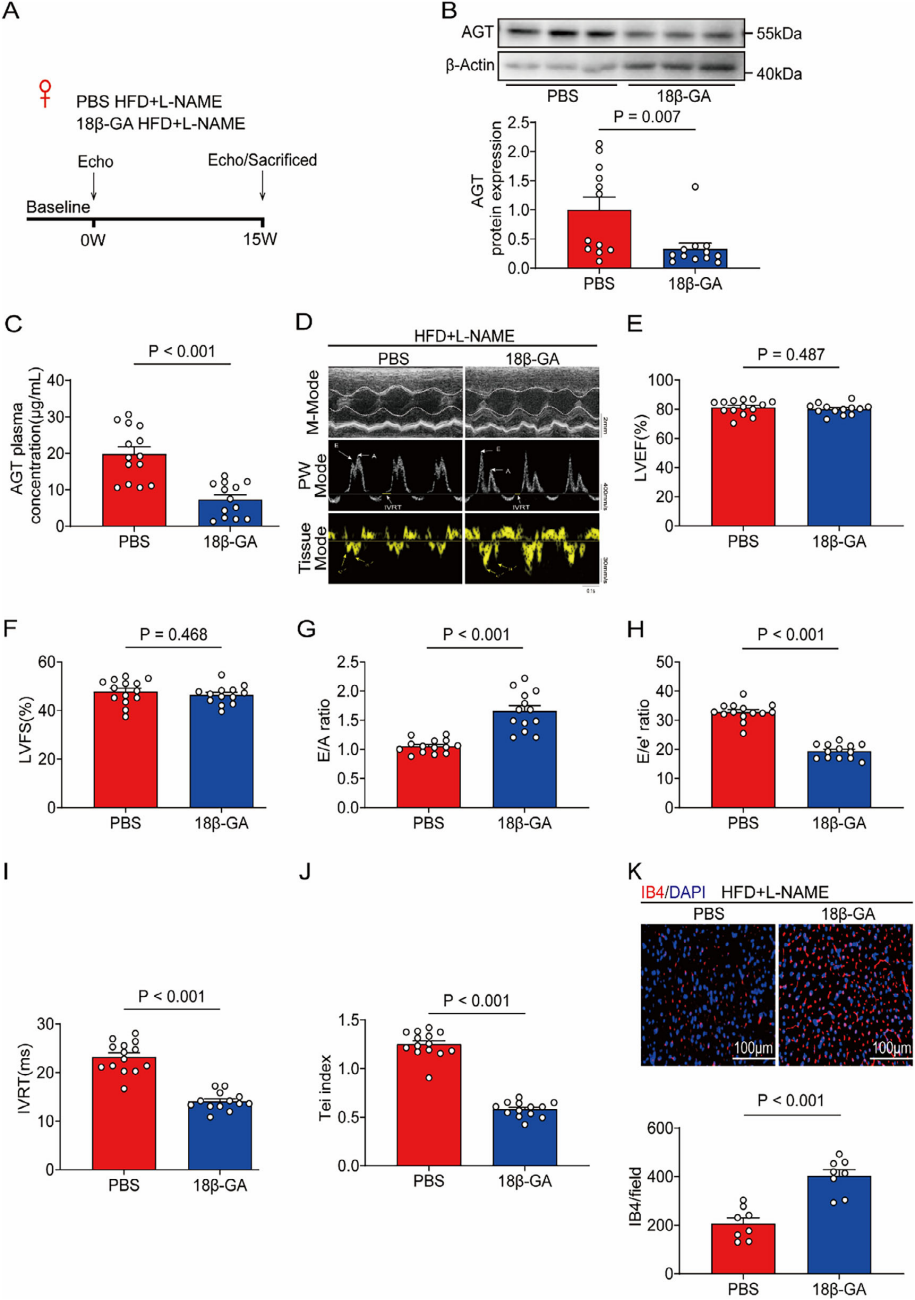
18β‐glycyrrhetinic acid (18‐βGA) is a potent suppressor of hepAGT to treat HFpEF in female mice. A) Experimental workflow and analysis for the effects of 18β‐GA treatment on HFpEF in female mice (n = 14 for PBS group, and n = 13 for 18β‐GA group). B) Western blotting detection and quantification of AGT protein abundances in hearts obtained from female HFpEF mice treated with 18β‐GA (n = 12 for each group). C) Plasma AGT concentrations were measured in female HFpEF mice treated with 18β‐GA (n = 14 for the PBS group, and n = 13 for the 18β‐GA group). D) Representative echocardiography images obtained from female HFpEF mice at 15w after treating with 18β‐GA (n = 14 for PBS group, and n = 13 for 18β‐GA group). E) Left ventricular ejection fraction (LVEF%) was quantified via echocardiography (n = 14 for PBS group, and n = 13 for 18β‐GA group). F) Left ventricular fraction shortening (LVFS%) was quantified via echocardiography (n = 14 for PBS group, and n = 13 for 18β‐GA group). G) E/A ratio was quantified via echocardiography (n = 14 for PBS group, and n = 13 for 18β‐GA group). H) E/e’ ratio was quantified via echocardiography (n = 14 for PBS group, and n = 13 for 18β‐GA group). I) IVRT was quantified via echocardiography (n = 14 for PBS group, and n = 13 for 18β‐GA group). J) Tei index was quantified via echocardiography (n = 14 for PBS group, and n = 13 for 18β‐GA group). K) Representative IB4 immune‐staining images of cardiac tissues obtained from female HFpEF mice at 15w after treating with 18β‐GA (n = 8 for each group, Bar = 100 µm). IB4 density of cardiac tissues was then calculated in female HFpEF mice at 15w after treating with 18β‐GA (n = 8 for each group). Mann‐Whitney U test was used for statistical analysis in B. Student's *t* test was used for statistical analysis in C–K.

Aiming to identify novel hepatic AGT inhibitors as potential therapeutic agents for HFpEF, we previously developed a promoter‐driven luciferase reporter system to enable high‐throughput screening of compounds targeting hepatic AGT production. 18β‐GA was then found to suppress hepAGT from 351 medicinal herb‐derived natural compounds.^[^ ^53^ ^]^ Based on our previous findings, we next investigated the effect of 18β‐GA on HFpEF. The female mice were treated with 18β‐GA, HFD, and L‐NAME simultaneously for 15 weeks (**Figure** **7**A). Hepatic AGT abundances and plasma AGT concentrations were decreased profoundly after 18β‐GA treatment (Figure 7B,C). Expectedly, 18β‐GA enhanced cardiac diastolic function (Figure 7D–J), promoted cardiac microvascular angiogenesis (Figure 7K). In addition, 18β‐GA treatment had no effect on hepatic and renal function (Figure S16A,B, Supporting Information). In conclusion, these data identified 18β‐GA as a promising hepAGT inhibitor to exert benefit to HFpEF.

## 3 Discussion

**Figure 8:**
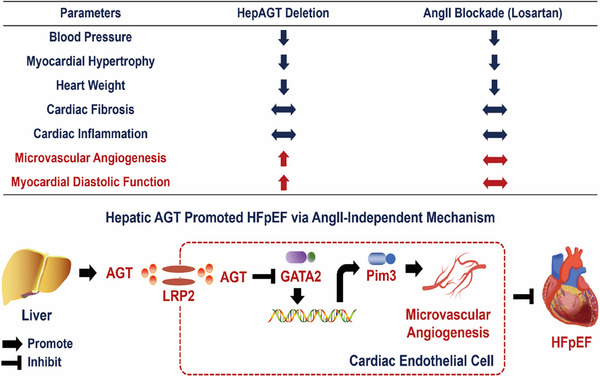
Potential mechanisms involved in hepatocyte‐derived angiotensinogen (hepAGT) contributing to HFpEF. Both interventions (hepatic AGT deletion vs systemic AngII blockade) showed similar outcomes in terms of blood pressure, myocardial hypertrophy, heart weight, cardiac fibrosis, and cardiac inflammation. However, they exhibited divergent effects on myocardial microvascular density and myocardial diastolic function in HFpEF models, irrespective of sex. These findings suggested that hepatic AGT may regulate HFpEF pathogenesis through an AngII‐independent mechanism centered on microvascular angiogenesis. Liver‐derived AGT was internalized by LRP2 in cardiac endothelial cells, subsequently contributing to myocardial diastolic dysfunction by suppressing microvascular angiogenesis via inhibiting the GATA2/Pim3 pathway. This cartoon illustration represents an original creation by our team.

In the current study, we demonstrated that hepatocyte‐derived AGT contributed to myocardial diastolic dysfunction in both female and male HFpEF mouse models. Further mechanistic study has revealed an AngII‐independent mechanism that participate in the hepAGT‐regulated HFpEF. We identified LRP2 as an original endocytic receptor for AGT internalization in cardiac endothelial cells. HepAGT is internalized by endothelial cells and then suppresses GATA2/Pim3 signal pathway, thereby inhibiting microvascular angiogenesis, ultimately promoting the development of HFpEF. 18β‐GA, a potent novel hepAGT suppressor, could effectively and safely improve cardiac diastolic function in HFpEF mice (**Figure** **8**).

### 3.1 The Unique Role of Hepatic AGT in HFpEF Pathogenesis

The RAS is a key system regulating blood pressure, fluid balance, and cardiovascular function. RAS inhibition is an important strategy for treating heart failure. In this regard, RAS inhibitors improve outcomes in HFrEF. In contrast, RAS blockers have provided disappointing results in HFpEF. Multiple pivotal large‐scale clinical trials, including CHARM‐Preserved,^[^ ^18^ ^]^ I‐Preserve,^[^ ^17^ ^]^ TOPCAT,^[^ ^16^ ^]^ PARAGON‐HF,^[^ ^54^ ^]^ and PEP‐CHF^[^ ^55^ ^]^ studies, have consistently demonstrated that RAS inhibitors fail to improve clinical outcomes (all‐cause mortality and heart failure hospitalization rates) in patients with HFpEF, highlighting the inherent limitations of these agents in HFpEF management. The following mechanisms may underlie this phenomenon: 1) Pathophysiological Heterogeneity: HFpEF pathogenesis involves multifaceted mechanisms such as chronic inflammatory activation, metabolic dysregulation (e.g., obesity, insulin resistance), myocardial hypertrophy, and microvascular dysfunction.^[^ ^30^, ^31^, ^32^, ^33^, ^34^, ^35^, ^36^, ^37^, ^38^, ^39^ ^]^ Conventional RAS inhibitors primarily target angiotensin II (AngII)‐mediated neurohormonal activation, which inadequately addresses this complex pathological network; 2) Differential RAS Activation: In contrast to HFrEF, where systemic RAS activation is prominent, HFpEF patients typically exhibit slight increased or normal systemic RAS activity, resulting in insufficient target engagement for RAS inhibitors; 3) The complexity of RAS components: It has been discovered that RAS not only includes the classic ACE‐AngII‐AT1R pathway, but also the ACE2‐Ang1‐7 pathway, renin‐PRR pathway, AngIV‐AT4R pathway, etc.^[^ ^56^, ^57^, ^58^, ^59^, ^60^ ^]^ These pathways formed complex crosstalk, and single inhibition of one component (e.g., ACE or AT1R) may lead to compensatory activation of other pathways,^[^ ^61^ ^]^ thereby diminishing therapeutic efficacy.

AGT is the sole precursor of RAS, synthesized by the liver, released into the circulation, and produces all active components of RAS.^[^ ^11^ ^]^ To date, studies have demonstrated that among components of the RAS, plasma AGT levels exhibit a significant and independent correlation with cardiac diastolic function.^[^ ^24^, ^25^ ^]^ This association persists even after adjusting for AngII‐dependent factors such as blood pressure and myocardial hypertrophy, suggesting that AGT may regulate diastolic function through AngII‐independent mechanisms. Interestingly, our current study has found that losartan, a systemic AngII blocker, cannot alleviate diastolic dysfunction in HFpEF mice, while hepatic deletion of AGT effectively improves diastolic function in HFpEF mice. These findings further support the potential of AGT as a therapeutic target for HFpEF via the AngII‐independent pathway.

### 3.2 HepAGT Regulates HFpEF via AngII‐Independent Mechanism

HFpEF involves multiple pathophysiological changes, including metabolic stress, cardiac hypertrophy, cardiac fibrosis, cardiac inflammation, and microvascular dysfunction.^[^ ^30^, ^31^, ^32^, ^33^, ^34^, ^35^, ^36^, ^37^, ^38^, ^39^ ^]^ The current study systematically investigated the impact of hepatic AGT knockout on these pathological pathways and revealed novel mechanistic insights into hepatic AGT‐driven HFpEF pathogenesis.

#### 3.2.1 Sex Difference of Metabolic Phenotypes after hepAGT Deletion in HFpEF Mice

Previous studies, including our own work, have reported the role and mechanisms of hepatic AGT in regulating obesity, insulin sensitivity, and lipid metabolism.^[^ ^28^, ^62^, ^63^, ^64^ ^]^ First, hepatic AGT knockout attenuated high‐fat diet (HFD)‐induced weight gain. Genetic rescue via intravenous administration of an adeno‐associated virus overexpressing des(AngI)AGT to hepAGT‐/‐ mice restored body weight gain to levels indistinguishable from HFD‐fed WT controls. This demonstrates hepatic AGT regulates weight gain independently of AngII. Subsequent microarray analysis revealed significantly elevated UCP1 and VEGF mRNA expression in brown adipose tissue of HFD‐fed hepAGT‐/‐mice versus HFD‐fed hepAGT+/+ mice, suggesting suppression of HFD‐induced weight gain may occur through enhanced thermogenesis and angiogenesis. Second, hepatic AGT knockout did not alter serum lipid profiles. However, it ameliorated HFD‐induced hepatic lipid deposition via activation of the AngII‐independent Akt/mTOR/SREBP1c signaling pathway. Finally, Hepatic AGT knockout improved insulin resistance. Given robust evidences that RAS inhibition, specifically targeting AngII, enhances insulin sensitivity,^[^ ^65^ ^]^ these data indicate hepatic AGT primarily regulates insulin resistance through an AngII‐dependent pathway.

In the current two‐hits (HFD + L‐NAME) HFpEF model, serum lipid levels remained unaltered in both female and male HFpEF mice following hepatic AGT knockout. However, hepatic AGT knockout attenuated weight gain and improved insulin resistance specifically in female HFpEF mice, with no such benefits observed in males. Notably, female HFpEF hepAGT‐/‐ mice fully recapitulated the metabolic phenotype (attenuated weight gain and improved insulin resistance) seen in hepAGT‐/‐ mice under HFD‐only conditions. This suggests conserved regulatory mechanisms in females – obesity attenuation via AngII‐independent pathways and insulin resistance improvement via AngII‐dependent pathways.

Previous studies have established that molecules associated with the nitric oxide (NO) signaling pathway (e.g., L‐citrulline) exhibit significant sex differences in their regulation of metabolic diseases, including obesity and diabetes. Moreover, this pathway critically modulates cardiac function.^[^ ^66^, ^67^ ^]^ As L‐NAME, a nitric oxide synthase (NOS) inhibitor, is a key pharmacological tool for modulating NO signaling, we hypothesize that the sex differences observed in hepatic AGT‐regulated metabolic phenotypes within the HFpEF model may be attributable to L‐NAME administration during model induction. Future investigations should employ high‐throughput technologies, such as metabolomics, to elucidate the specific underlying molecular mechanisms.

#### 3.2.2 Hepatic AGT Deficiency Did Not Alter Cardiac Inflammation and Fibrosis in HFpEF Mice

Cardiac inflammation and fibrosis are key pathogenic factors in HFpEF. Inflammatory factor release activates fibrotic signaling pathways, increasing cardiac stiffness and ultimately causing diastolic dysfunction.^[^ ^68^, ^69^, ^70^ ^]^ Previous human tissue studies demonstrate that HFpEF patients display mildly elevated cardiac fibrosis compared to healthy controls. Notably, no significant difference exists in cardiac fibrosis severity between HFrEF and HFpEF patients, indicating that non‐fibrotic mechanisms contribute to HFpEF pathogenesis.^[^ ^35^ ^]^

In this study, hepatic AGT‐knockout mice showed no significant alterations in cardiac tissue mRNA expression of inflammatory/fibrotic markers, compared to their hepAGT+/+ littermates. These findings demonstrate that hepatic AGT does not modulate cardiac inflammation/fibrosis in HFpEF. Hepatic AGT regulates HFpEF through mechanisms independent of inflammation and fibrosis.

#### 3.2.3 Hepatic AGT Knockout versus Systemic AngII Inhibition Showed Comparable Outcomes in Terms of Cardiac Hypertrophy

AngII is the main bioactive peptide of RAS and contributes to heart failure by elevating blood pressure and leading to cardiac hypertrophy.^[^ ^71^, ^72^, ^73^ ^]^ In the HFpEF model used in this study, we simulated the characteristic hypertensive phenotype of human HFpEF by administering L‐NAME. This intervention induced hypertension‐associated cardiac alterations, including increased heart weight, myocardial hypertrophy, and enlarged cardiomyocyte size. Both hepatic AGT knockout and losartan‐mediated inhibition of the RAS effectively attenuated these structural changes, reducing heart weight, decreasing cardiomyocyte size, and ameliorating myocardial hypertrophy. These results indicate that hypertension‐driven cardiac structural remodeling in this HFpEF model primarily originates from pathological activation of the classic RAS pathway.

Notably, however, while hepatic AGT knockout significantly improved cardiac diastolic function in HFpEF mice, losartan treatment did not improve diastolic function. This discrepancy implies the involvement of additional mechanisms beyond cardiac hypertrophy regulation in hepatic AGT‐mediated HFpEF pathology.

#### 3.2.4 Microvascular Angiogenesis: A Bridge Connecting hepAGT and HFpEF

Impaired myocardial angiogenesis is now recognized as a significant contributor to HFpEF pathogenesis.^[^ ^35^ ^]^ We therefore investigated the role of angiogenesis in hepatic AGT‐mediated HFpEF regulation. Previous studies established associations between AGT and angiogenesis. AGT is cleaved by renin into AngI and des(AngI)AGT, with des(AngI)AGT constituting 97% of circulating AGT. In vitro studies demonstrate that des(AngI)AGT inhibits angiogenesis, indicating AngII‐independent angio‐regulation by AGT.^[^ ^28^, ^74^ ^]^

In the current study, hepatic AGT deletion significantly increased myocardial capillary density – but not arteriolar density – in both male and female HFpEF mice. Systemic AngII blockade (via losartan) did not affect capillary density in either sex. Mechanistically, hepatic AGT deficiency promoted angiogenesis through GATA2/Pim3 pathway activation. These findings establish hepatic AGT as an AngII‐independent regulator of myocardial angiogenesis in HFpEF.

The potential mechanisms linking cardiac microvascular rarefaction to cardiac stiffness and diastolic dysfunction include: 1) Myocardial Energetic Impairment: Reduced perfusion compromises myocardial energy synthesis, impairing cytosolic Ca2+ clearance, prolonging myocardial relaxation, and contributing to diastolic dysfunction;^[^ ^48^ ^]^ 2) Myocardial Fibrosis: Microvascular rarefaction decreases local nitric oxide (NO) synthesis and increases endothelin release, activating fibroblasts into myofibroblasts, promoting collagen deposition, and elevating myocardial stiffness;^[^ ^35^, ^46^, ^47^ ^]^ 3) Cardiomyocyte Calcium Dysregulation: Hypoxia and oxidative stress associated with rarefaction impair calcium handling, leading to diastolic calcium overload and dysfunction;^[^ ^47^, ^75^, ^76^, ^77^ ^]^ 4) Inflammatory Response: Ischemia and hypoxia trigger inflammatory cytokine release (e.g., IL‐1β, TNF‐α), activating fibrotic pathways that further compromise diastolic function;^[^ ^47^ ^]^ 5) Reduced Diastolic Coronary Perfusion: Microvascular rarefaction diminishes coronary reserve, compromises diastolic myocardial perfusion, disrupts ventricular‐arterial coupling, and hinders effective ventricular filling.^[^ ^78^ ^]^

Given the absence of hepatic AGT effects on cardiac inflammation and fibrosis in HFpEF mice, and our previous finding that hepAGT impairs cardiac function via calcium dysregulation in acute cardiac injury models,^[^ ^45^ ^]^ we assessed calcium‐handling proteins. Hepatic AGT‐knockout (hepAGT‐/‐) HFpEF mice exhibited significantly increased phospholamban phosphorylation (elevated p‐PLN/PLN ratio) without altered SERCA2a expression. Furthermore, specific knockdown of LRP2 in cardiac endothelial cells to block hepatic‐derived AGT entry into the heart, followed by HFpEF induction, similarly elevated cardiac PLN phosphorylation levels without significantly altering SERCA2a or titin expression. These findings indicate that enhanced cardiomyocyte calcium handling underlies the improved diastolic function following hepatic AGT deletion, likely consequent to attenuated microvascular rarefaction.

### 3.3 LRP2: A Novel Receptor for hepAGT Internalization to Regulate Cardiac Angiogenic Responses in HFpEF

AGT synthesized by the liver is released into the circulation and can be internalized in non‐cardiomyocytes in cardiac tissues. In a model of sepsis‐induced cardiac dysfunction (SIMD), hepAGT is primarily taken up by cardiac fibroblasts through low‐density lipoprotein receptor‐related protein 1 (LRP1), thereby mediating the development of SIMD.^[^ ^45^ ^]^ Interestingly, we have also identified a similar “liver‐heart crosstalk” mechanism in the HFpEF model. In the current study, we found that LRP2 is highly abundant in cardiac microvascular endothelial cells. By inhibiting LRP2 expression in cardiac endothelial cells in vivo, the abundance of cardiac AGT was significantly downregulated, and cardiac diastolic function markedly improved. The phenomenon of exogenous AGT internalization mediated by LRP2 has previously been validated mainly in renal diseases, where it primarily occurs in renal proximal tubule cells.^[^ ^79^, ^80^, ^81^ ^]^ However, the current study demonstrates that this phenomenon also exists in cardiac microvascular endothelial cells and participates in the regulation of HFpEF by inhibiting angiogenic signaling pathways. These findings not only provide new evidence for the regulatory role of AGT‐mediated liver‐heart interactions in HFpEF, but also provide insight into how the same ligand might play different roles, depending on the differential expressions of receptors in diverse tissues.

### 3.4 The Perspective of Targeting Hepatic AGT to Treat HFpEF

Targeted suppression of hepatic AGT currently relies primarily on RNA interference (RNAi) strategies, including antisense oligonucleotides (ASOs) and small interfering RNAs (siRNAs). These approaches utilize GalNAc conjugation technology to achieve > 90% liver‐targeted delivery, significantly minimizing off‐target effects.^[^ ^82^, ^83^, ^84^ ^]^ Multiple large‐scale randomized controlled trials (RCTs) have confirmed that RNAi‐based strategies safely and effectively reduce plasma AGT levels. Their low‐frequency dosing regimen (e.g., biannual administration) enables sustained blood pressure reduction,^[^ ^85^, ^86^, ^87^ ^]^ they also enhance compliance. Collectively, these findings validate the efficacy and safety of RNAi‐mediated hepatic AGT suppression.

Compared to RNAi and gene editing technologies, small‐molecule compounds offer distinct advantages: 1) absence of immune activation, 2) lower production costs, and 3) well‐characterized target engagement with minimized off‐target effects.^[^ ^88^ ^]^ Capitalizing on these benefits, our current work also identified the small‐molecule compound 18β‐glycyrrhetinic acid (18β‐GA), which targets hepatic AGT by downregulating hepatocyte nuclear factor 4α (HNF4α), consequently reducing plasma AGT concentrations.^[^ ^53^ ^]^

Given our discovery that hepatic AGT represents an effective therapeutic target for HFpEF intervention, we propose that AGT suppression via either RNAi technology or small‐molecule compounds (e.g., 18β‐GA) holds promise as a novel therapeutic strategy for HFpEF.

### 3.5 Study Limitations

This study has several limitations. First, invasive hemodynamic assessments of diastolic function (e.g., pressure‐volume loop‐derived tau) were not performed due to equipment constraints. Second, while investigation of compensatory mechanisms mediated by tissue‐specific RAS (e.g., cardiac, renal, cerebral) in HFpEF pathogenesis would require mouse models with conditional AGT deficiency in specific cell types (such as MCECs, renal tissue, or brain tissue), such tissue‐specific knockout models have not been optimized. Third, this study utilized the most commonly employed “high‐fat diet + L‐NAME” two hits protocol to establish an HFpEF model. While this approach partially recapitulates key phenotypes observed in clinical HFpEF patients – namely hypertension and metabolic dysfunction – it does not fully replicate the spectrum of clinical characteristics found in this patient population. Fourth, observed sex‐specific differences in metabolic phenotypes following hepatic AGT deletion in HFpEF models necessitate future metabolomics for mechanistic elucidation. Finally, the reliance on murine models introduces limitations, including potential differences in RAS regulatory mechanisms between mice and humans, raising questions about translational relevance.

## 4 Conclusion

The current study demonstrated that increased hepatic AGT abundance led to cardiac diastolic dysfunction via the AngII‐independent pathway. Liver‐derived AGT was internalized by LRP2 in cardiac endothelial cells, subsequently contributing to myocardial diastolic dysfunction by suppressing microvascular angiogenesis via inhibiting the GATA2/Pim3 pathway. Additionally, in vivo 18β‐GA infusion may be a promising approach targeting hepAGT for HFpEF therapy.

## 5 Experimental Section

### Experimental Design

This study aims to determine the effect of hepatic AGT on HFpEF and its underlying mechanisms. Through molecular biological techniques, Western blotting, and tissue bulk RNA sequencing, the comprehensive mechanisms by which AGT regulated HFpEF were explored. All mice used in the current study were randomly assigned to experimental groups based on a randomization list generated by PASS 11.0 software (NCSS, UT, USA). Various measurements and calculations were performed by researchers who were blinded to the group and treatment information, including echocardiographic measurements and image acquisition, immunofluorescence image collection, and counting.

### Animals

All mice were maintained in a barrier facility under a 12‐h light/12‐h dark cycle (environmental temperature at 23 °C) and were fed standard laboratory chow. All mouse experiments were approved by the Animal Care and Use Committee of Zhejiang University (Number AIRB‐2022‐0257) and adhered to the guidelines for the care and use of laboratory animals established by the National Institutes of Health. AGT‐floxed mice and albumin‐Cre transgenic mice were generated by the team. HepAGT‐deficient mice (hepAGT‐/‐) were produced by mating AGT‐floxed mice with albumin‐Cre transgenic mice. Mice that did not carry the albumin‐Cre transgene (hepAGT+/+) served as the control group.

The primers used for genotyping were listed as follows:

LoxP:

F 5′‐AACCTTGTCTGGAGTGGG‐3′,

R 5′‐TCAGAGATCCGTGGGAAC‐3′;

Albumin‐Cre:

F 5′‐ACCTGAAGATGTTCGCGATT‐3′,

R 5′‐CGGCATCAACGTTTTCTTTT‐3′.

### Establishment of HFpEF Mouse Models

The HFpEF model in the current study was established by feeding C57BL/6N mice with a high‐fat diet (HFD) (#D12492, Research Diets, USA) in which 60% of the calories were provided by fat and 0.5 g L^−1^ of Nω‐Nitro‐L‐arginine methyl ester hydrochloride (L‐NAME) (#N5751, Sigma–Aldrich, USA) in the drinking water for 15 weeks. Mice aged 8–10 weeks with good nutritional status were bred and randomly grouped. Before model establishment, measurements including blood pressure, echocardiography, and body weight were completed.

### Protocol for Mouse Echocardiography

One day prior to the examination, remove hair from the chest and subxiphoid region of the mice using depilatory cream for skin preparation. On the day of imaging, anesthetize the mice in a gas anesthesia system by placing them in an induction chamber with 5% isoflurane. Once the mice exhibit no response to hind‐limb pinch reflexes, transfer them to the ultrasound platform and secure them in a supine position. Attach gel‐based electrocardiogram (ECG) electrodes to all four limbs for heart rate monitoring. Maintain anesthesia via a nose cone connected to a rubber anesthesia hose delivering 1–2% isoflurane, ensuring a stable heart rate of 400–500 beats per minute. Apply ultrasonic coupling gel to the chest and gently position the MS400 ultrasound probe to acquire standard long‐axis and short‐axis views of the left ventricle. Use M‐mode echocardiography to measure systolic function parameters, including Left Ventricular Ejection Fraction (LVEF) and Left Ventricular Fractional Shortening (LVFS). Subsequently, obtain a four‐chamber view to assess diastolic function, including the peak early diastolic filling velocity (E peak), peak late diastolic filling velocity (A peak), early diastolic mitral annulus velocity (e’ peak), isovolumic relaxation time (IVRT), isovolumetric contraction time (IVCT), and ejection time (ET). Tei index was calculated as the ratio of IVCT and IVRT divided by ET.

### Mouse Tissues Collection

Mice were anesthetized via intraperitoneal injection of sodium pentobarbital solution (100–200 mg kg^−1^) until there was no response to pressure on the hind limbs. The mice were then carefully secured on the surgical table. Using curved forceps, the abdominal skin was lifted, and a V‐shaped incision was made along the midline of the abdomen. The diaphragm was cautiously incised along the rib margins using ophthalmic scissors, and the surrounding adipose tissue was removed through blunt dissection to fully expose the heart. A sterile syringe, lubricated with a coagulant, was gently inserted into the right ventricle from the right side of the apex, and blood was slowly drawn. The collected blood was transferred into a coagulant tube and allowed to sit on ice before being centrifuged at 5000 rpm for 15 min at 4 °C. The plasma was then collected into a new EP tube, properly labeled, and stored at −80 °C for future use. Subsequently, the liver, heart, and tibia were isolated from the mice. The blood within the heart was flushed out using PBS, and after blotting with absorbent paper to remove the PBS, the weight of the heart was measured. Depending on the requirements, the heart was then placed in 4% paraformaldehyde fixative or liquid nitrogen. After thoroughly separating the muscle and other tissues from the tibia, the length of the tibia was measured using a caliper. Other organs, such as the liver, were similarly placed in either 4% paraformaldehyde fixative or liquid nitrogen as needed.

### Intraperitoneal Glucose Tolerance Test (IPGTT)

Mice were fasted overnight for 16 h, followed by measurement of fasting blood glucose levels via the tail vein. Next, normal saline containing 20% (w/v) glucose (2g glucose per kilogram body weight) was intraperitoneally injected into each mouse, and blood samples were collected at 15, 30, 60, and 120 min after injection for further blood glucose measurement.

### Plasma ELISA

The concentrations of mouse AGT (#ab245718, Abcam, USA), Ang II (Angiotensin II; #ADI‐900‐204, Enzo Life Sciences, Inc., USA), total triglyceride(#A110‐1‐1, Nanjing Jiancheng Bioengineering Institute, China), total cholesterol(#A111‐1‐1, Nanjing Jiancheng Bioengineering Institute, China), alanine aminotransferase (#C009‐2‐1, Nanjing Jiancheng Bioengineering Institute, China), aspartate aminotransferase (#C010‐2‐1, Nanjing Jiancheng Bioengineering Institute, China), and creatinine (#C011‐2‐1, Nanjing Jiancheng Bioengineering Institute, China) in plasma were measured using ELISA kits, following the manufacturer's instructions.

### The Preparation of Paraffin Tissue Specimens and Masson's Trichrome Staining

Fix the tissue in 4% paraformaldehyde solution for 24 h. Following fixation, rinse the tissue block thoroughly under running water and transfer it to an automated tissue processor for dehydration. Upon completion of dehydration, immerse the tissue in molten paraffin for infiltration. Select an appropriate embedding mold, carefully position the paraffin‐infiltrated tissue block at the center of the mold, and pour molten paraffin into the mold to fully encapsulate the tissue. Transfer the mold to a cooling platform and allow the paraffin to solidify completely. Once solidified, gently remove the paraffin‐embedded tissue block using a small spatula. Trim the paraffin block to an optimal shape with a trimming knife and mount it onto a paraffin microtome. Gradually trim the block incrementally until the tissue surface was adequately exposed. Set the microtome to a sectioning thickness of 5 µm and cut 10 consecutive tissue sections. Float the sections on a 40 °C warm water bath to ensure proper flattening. Collect the sections using adhesive‐coated glass slides, ensuring sufficient tissue coverage (typically two sections per slide). Dry the slides on a slide warmer at 65 °C for 1–2 h. Once dried, store the slides in a slide box at room temperature for subsequent use. For staining, follow the manufacturer's protocol using the Masson staining kit (#G1346, Solarbio, China) to process the dried paraffin sections.

### Immunofluorescence

The harvested cardiac tissues were dehydrated in 30% sucrose solution for 24 h, embedded in optimal cutting temperature (OCT) compound (#BL1674A, Biosharp, China), and frozen at −80 °C. Serial sections of 6 µm thickness were prepared using a cryostat. Sections were fixed with 4% paraformaldehyde (PFA) for 15 min, permeabilized with 0.1% Triton X‐100 for 10 min, and blocked with 5% bovine serum albumin (BSA) in phosphate‐buffered saline (PBS) for 1 h at room temperature. Primary antibodies diluted in blocking buffer were applied overnight at 4 °C:

Anti‐LRP1 (Rabbit monoclonal, #ab92544, Abcam, 1:100);

Anti‐LRP2 (Rabbit polyclonal, #19700‐1‐AP, ProteinTech Group, 1:250);

Anti‐α‐smooth muscle actin (α‐SMA) (Mouse monoclonal, #ab7817, Abcam, 1:400);

Anti‐IB4 (#I21412, Thermo Scientific, 1:200);

Anti‐SERCA2a (Rabbit monoclonal, #9850S, Cell Signaling Technology, 1:50);

Anti‐Titin (Rabbit polyclonal, #27867‐1‐AP, ProteinTech Group, 1:400).

The following day, sections were incubated with species‐matched fluorescent secondary antibodies for 1 h at room temperature:

Donkey anti‐rabbit IgG (H&L) (DyLight® 488, #ab96891, Abcam, 1:500) for LRP1 and LRP2;

Donkey Anti‐Mouse IgG H&L (DyLight® 488, #ab98794, Abcam, 1:500) for α‐SMA;

The IB4 antibody exhibits intrinsic fluorescence, thus fluorescent secondary antibodies were not required;

Anti‐WGA (Wheat Germ Agglutinin, #W32464, Invitrogen, 1:500) was incubated at room temperature for 15 min.

Nuclei were counterstained with 4′,6‐diamidino‐2‐phenylindole (DAPI, 100 ng mL^−1^) for 5 min. For quantification, IB4‐positive and α‐SMA‐positive cells were counted in 3–4 sections per heart using ImageJ software, with 10 randomly selected high‐power fields (400×magnification) analyzed per section.

### Losartan Administration

Losartan (#L335345, Aladdin, China) was dissolved in PBS and administered to mice via drinking water at a concentration of 0.2 g L^−1^. An equal volume of PBS was added to the drinking water of the control group.

### 18β‐GA Administration

Female C57BL/6 mice (weighing 20–22 g) were intraperitoneally injected with 50 mg kg^−1^ of 18β‐GA (#DG0007, Chengdu DeSiTe Biological Technology, China). This compound was dissolved in PBS, and the injections were administered every other day for 15 weeks.

### Blood Pressure Measurement

Blood pressure was monitored in mice using a non‐invasive tail‐cuff system (BP‐2000, Visitech Systems, Apex, NC, USA). Mice were acclimatized to the procedure through a 5‐day training regimen prior to final measurements. On the day of data collection, systolic and diastolic blood pressure values were recorded as the average of 20 consecutive measurements per mouse. To ensure accuracy, waveform morphology and pressure tracings were manually verified, and readings with motion artifacts or irregular pulse patterns were excluded.

### Cell Culture

Mouse cardiac endothelial cells (MCECs) (#BNCC359855, BeNa Culture Collection) were cultured in high glucose DMEM (#11 965 092, Thermo Scientific, USA) supplemented with 10 % fetal bovine serum (#35–081‐CV, CORNING, USA) with 1 % antibiotics (100 U/mL penicillin and 100 µg mL^−1^ streptomycin) at 37 °C and 5 % CO2.

### Isolation and Culture of Mouse Hepatocytes

Under anesthesia, the inferior vena cava and portal vein of mice were surgically exposed. The liver was perfused retrograde through the portal vein with sequential solutions: Perfusion Phase 1: 20 mL of ice‐cold Dulbecco's Hank's Balanced Salt Solution (D‐HBSS) to clear residual blood;Perfusion Phase 2: 50 mL of pre‐warmed (37 °C) digestion solution containing 0.85 mg mL^−1^ Collagenase Type II (#17 101 015, Gibco, USA). The digested liver was excised, rinsed in Dulbecco's Modified Eagle Medium (DMEM) supplemented with 10% fetal bovine serum (FBS), and mechanically dissociated by tearing the liver capsule to release hepatocytes. The cell suspension was filtered through a 100 µm cell strainer and centrifuged at 50×g (4 °C, 2 min) to pellet hepatocytes. The pellet was resuspended in DMEM + 10% FBS and subjected to two additional low‐speed centrifugation cycles (50×g, 4 °C, 2 min each) to remove non‐parenchymal cells and debris.

Purified hepatocytes were resuspended in DMEM + 10% FBS and seeded onto rat tail collagen‐coated culture plates. After 5 h of adhesion, the medium was replaced with serum‐free DMEM. Primary hepatocytes were cultured overnight (37 °C, 5% CO2), and conditioned supernatant was collected for further analysis.

### Separation of Non‐Cardiomyocyte Cell Populations using Fluorescence Activated Cell Sorting

Heart tissues were subjected to enzymatic digestion using 2 mg mL^−1^ collagenase II (#17 101 015, Gibco, USA) at 37 °C for 1 h, followed by gentle mechanical dissociation for 2 min. After two additional digestion cycles, the cell suspension was centrifuged (300×g, 5 min), and the resulting pellet was washed twice with FACS buffer (0.5% FBS in phosphate‐buffered saline, PBS). The pellet was resuspended in 3 mL of red blood cell lysis buffer (#R1010, Solarbio, China), vortexed briefly, and incubated for 2 min at room temperature to facilitate lysis. The reaction was halted by adding an equal volume of FACS buffer, followed by centrifugation (300×g, 5 min). The pellet was washed once with 5 mL of FACS buffer and centrifuged under identical conditions.

For flow cytometry staining, cells were pre‐treated with Fc receptor blocking solution (1:100 dilution, #553 141, BD Pharmingen, USA) on ice for 5 min to minimize nonspecific binding. Subsequently, cells were incubated with fluorophore‐conjugated antibodies at room temperature for 15 min: CD45 (1:200, rat anti‐mouse, #567 659, BD Pharmingen, USA), CD31 (1:200, rat anti‐mouse, #561 410, BD Pharmingen, USA), CD140a (PDGFRα) (1:200, rat anti‐mouse, #562 777, BD Pharmingen, USA).

Stained cells were sorted on a BD FACS Aria II flow cytometer into three gated populations: CD45^−^ PDGFRα⁺ cardiac fibroblasts (CFs), CD45⁺ immune cells, CD45^−^ CD31⁺ endothelial cells. Sorted cell populations were collected for further RNA extraction.

### Tube Formation Assay

MCECs were preconditioned under experimental treatments for 24 h. Pre‐chilled 96‐well plates were coated with 50 µL of growth factor‐reduced Matrigel (#356 231, Corning, USA) on ice and polymerized at 37 °C for 40 min. After gel solidification, 5×10⁴ MCECs per well were suspended in 100 µL of their respective experimental medium and seeded onto the Matrigel‐coated plates. Cells were incubated for 6 h under standard culture conditions to allow capillary‐like network formation. Three independent fields of view per well were imaged using an inverted phase‐contrast microscope (Leica Microsystems, Germany). Total tube length per field was quantified using ImageJ software (v1.53, NIH, USA).

### SiRNA Transfection

MCECs were transfected with siRNA targeting LRP2 mRNA or scrambled control siRNA using Lipofectamine RNAiMAX Transfection Reagent (#13 778 500, Invitrogen), following the manufacturer's protocol. Briefly: 50 nm siRNA was complexed with 4 µL Lipofectamine RNAiMAX in Dulbecco's Modified Eagle Medium (DMEM) and incubated at room temperature for 20 min. The siRNA‐lipid complex was added to MCECs and incubated for 24 h in DMEM containing 0.5% FBS. After 24 h, the transfection medium was replaced with fresh DMEM. Cells were cultured for an additional 48 h (72 h total post‐transfection) prior to stimulation with experimental agonists for downstream assays.

### Lentivirus Transfection

MCECs were transfected with lentiviruses carrying shRNA targeting Pim3 or GATA2 mRNA or scrambled control shRNA. Lentivirus was added to MCECs at a titer of 50 MOI for incubation. After 8 h, the transfection medium was replaced with fresh DMEM, and the cells were cultured for another 24 h.

### Recombinant Angiotensinogen Protein Treatment

Recombinant angiotensinogen protein (His‐Tag AGT) (#55337‐M08H, Sino Biological, China) at a concentration of 20 nmol L^−1^ or an equal volume of PBS was added to the MCECs and incubated for 24 h. After incubation, the culture dish was rinsed with sterile PBS to prepare for protein extraction.

### Adeno‐Associated Viruses (AAVs) Construction and Myocardial Injection

AAV vectors with endothelial‐specific promoter Tie2 encoding short hairpin RNA (shRNA) targeting LRP2 were designed and packaged by Shanghai GeneChem Co., Ltd. (China). Viral particles were amplified in HEK293T cells following the manufacturer's protocol, purified, and quantified via qPCR to achieve a final titer of 1 × 10⁹ transducing units (TU)/mL. AAVs harboring scrambled shRNA sequences served as negative controls.

For in vivo delivery, mice were anesthetized, and the thoracic cavity was surgically opened to expose the heart. AAVs carrying LRP2 shRNA or scrambled shRNA constructs (3 × 10^1^ ^2^ viral genome copies per mouse) were injected into five sites across the left ventricular anterior wall using a 50 µL Hamilton microsyringe (#705, USA). Each injection site received 8 µL of viral suspension (40 µL total per heart) via direct myocardial puncture.

### Dual‐Luciferase Reporter Assay

For the dual luciferase reporter gene assay, a dual luciferase vector with the GATA2 promoter was constructed using the GPL4 vector. HEK293T cells were cultured and co‐transfected with either the GPL4‐Pim3 wild‐type (GPL4‐Pim3 promoter WT) or the GPL4‐Pim3 mutant (GPL4‐Pim3 promoter MUT) along with the GATA2‐pcDNA plasmid. All cells were washed, and the luciferase activity in the cell lysates was measured using a dual luciferase reporter assay kit (#G06001, Gene Pharma), following the manufacturer's instructions.

### RNA‐sequencing and Data Analysis

HepAGT+/+ and HepAGT‐/‐ mice were fed with a high‐fat diet (HFD) (#D12492, Research Diets, USA) and Nω‐Nitro‐L‐arginine methyl ester hydrochloride (L‐NAME) (#N5751, Sigma–Aldrich, USA), and cardiac tissue was harvested. Total RNA of heart tissues was extracted by TRIzol reagent (#15 596 018, Life Technologies) for RNA‐sequencing (three biological replicates for each group).

RNA‐sequencing was performed by Novogene (Beijing, China). Briefly, total RNA was purified from total RNA using poly‐T oligo‐attached magnetic beads. Sequencing libraries were generated using NEBNext® UltraTM RNA Library Prep Kit for Illumina (NEB, USA) following the manufacturer's recommendations, and index codes were added to attribute sequences to each sample. The clustering of the index‐coded samples was performed on a cBot Cluster Generation System using TruSeq PE Cluster Kit v3‐cBotHS (Illumia) according to the manufacturer's instructions. After cluster generation, the library preparations were sequenced on an Illumina Hiseq platform, and 150 bp paired‐end reads were generated. For data analysis, raw data (raw reads) in fastq format were first processed through in‐house Perl scripts. Clean data (clean reads) were obtained by removing reads containing adapters and ploy‐N as well as low‐quality reads from raw data. Reference genome and gene model annotation files were downloaded from the genome website directly. An index of the reference genome was built using STAR, and paired‐end clean reads were aligned to a reference genome using STAR (v2.5.1b). STAR uses the method of Maximal Mappable Prefix. HTSeq v0.6.0 was used to count read numbers mapped to each gene. Analysis of differential expression was performed using the edgeR R package (3.12.1). P values were adjusted using the Benjamini and Hochberg method. GO and Kyoto Encyclopedia of Genes and Genomes pathway analyses were implemented using the clusterProfiler R package. The hierarchical clustering heat map was generated with the ggplot library.

### Quantitative Polymerase Chain Reaction

Total mRNA was extracted from the liver and heart tissues by TRIzol reagent (#15 596 018, Invitrogen, USA) and reverse‐transcribed to cDNA using PrimeScript RT Reagent Kit (#RR037A, Takara, Japan). The quantitative polymerase chain reaction was performed by TB Green® Premix Ex Taq RT‐PCR kit (#RR420A, Takara, Japan) on Applied Biosystems 7500 Fast Real‐Time PCR System (ABI, Torrance, CA). The quantitative polymerase chain reaction program was conducted with initial denaturation (95 °C for 30 s) followed by 40 cycles consisting of denaturation (95 °C for 5 s) and anneal/extension (60 °C for 32 s). The mRNA abundances of target genes were normalized to β‐actin and calculated via the standard 2‐△△Ct method. The sequences of primers used in the current study are listed as follows:

**Table:** 

Gene	Forward sequence (5′→3′)	Reverse sequence (5′→3′)
β‐actin	AGATCAAGATCATTGCTCCTCCT	ACGCAGCTCAGTAACAGTCC
ANP	TCGTCTTGGCCTTTTGGCTT	GTGGTCTAGCAGGTTCTTGAAAT
BNP	GCCAGTCTCCAGAGCAATTCA	ACAACAACTTCAGTGCGTTACA
Col1α2	GCTCCAAAGGAGAATCCGGT	TTGCCAGGAGGACCCATTAC
Col1α1	TGACTGGAAGAGCGGAGAGTA	CAGACGGCTGAGTAGGGAAC
Col3α1	AAGGCTGCAAGATGGATGCT	GAGGGCCATAGCTGAACTGAA
TGF‐β	GATACGCCTGAGTGGCTGTC	GTTTGGGGCTGATCCCGTTG
Postn	CCTGCCCTTATATGCTCTGCT	AAACATGGTCAATAGGCATCACT
Fn1	GCCGTTAGATGTGCAAGCTG	TGCTGAAGCTGAGAACTAGGC
ACTA2	GGACGTACAACTGGTATTGTGC	TCGGCAGTAGTCACGAAGGA
TNF‐α	CATGAGCACAGAAAGCATGATCCG	AGCAGGAATGAGAAGAGGCTGAG
IL‐6	ACAACCACGGCCTTCCCTACTT	CACGATTTCCCAGAGAACATGTG
IL‐1β	CCCAACTGGTACATCAGCAC	TCTGCTCATTCACGAAAAGG
LRP1	CTGGCGTGGTGTTCTGGTAT	TTGGTAGGCTTGTCAGGGTC
LRP2	AAAATGGAAACGGGGTGACTT	GGCTGCATACATTGGGTTTTCA

### Western Blotting

Tissues or cells were lysed in RIPA lysis buffer (25 mm Tris‐HCl pH 7.6, 150 mm NaCl, 1% NP‐40, 0.5% sodium deoxycholate, 0.1% SDS) on ice. Total protein concentrations were quantified using a BCA assay, and equal amounts of protein (20–50 µg per lane) were resolved by SDS‐PAGE on 8%, 10%, or 12% acrylamide gels. Electrophoresis was performed in 1×running buffer (25 mm Tris base, 192 mm glycine, 0.1% SDS) at 100 V for 90–120 min. Proteins were then transferred to polyvinylidene difluoride (PVDF) membranes using a wet transfer system in 1×transfer buffer (25 mm Tris, 192 mm glycine, 20% methanol) at 300 mA for 90 min.

Membranes were blocked with 5% non‐fat milk in TBST (Tris‐buffered saline with 0.1% Tween‐20) for 1 h at room temperature and subsequently incubated overnight at 4 °C with primary antibodies diluted in blocking buffer according to the manufacturer's specifications. After three washes with TBST, membranes were probed with horseradish peroxidase (HRP)‐conjugated secondary antibodies for 1 h at room temperature. Protein bands were visualized using Immobilon Western Chemiluminescent HRP Substrate and imaged on a ChemiDoc Imaging System (Bio‐Rad, USA). Signal intensities were quantified using Image Lab Software.

The detailed information of antibodies used in the study was listed as follows:

β‐actin: #KC‐5A08, Aksomics Inc., 1:3000, predicted molecular weight: 42 kDa;

AGT: #28 101, Immuno‐Biological Laboratories, 1:100, bands can be detected within 47.3–84.7 kDa, predicted molecular weight: 53 kDa;

LRP2: #19700‐1‐AP, ProteinTech Group, 1:1000, predicted molecular weight: 280 kDa;

His‐tag: #ab9108, Abcam, 1:1000;

Pim3: #ab198842 Abcam, 1:1000, predicted molecular weight: 37kDa;

GATA2: #ab109241 Abcam, 1:1000, predicted molecular weight: 51kDa;

SERCA2a: #9580S, Cell Signaling Technology, 1:1000, predicted molecular weight: 114, 140 kDa;

p‐PLN: #HA722291, HUABIO, 1:1000, predicted molecular weight: 12 kDa;

PLN: #HA500103, HUABIO, 1:1000, predicted molecular weight: 12 kDa;

Horseradish peroxidase‐labeled goat anti‐rabbit IgG(H+L): #A0208, Beyotime Biotechnology, 1:1000;

Horseradish peroxidase‐labeled goat anti‐mouse IgG(H+L): #A0216, Beyotime Biotechnology, 1:1000;

Horseradish peroxidase‐labeled donkey anti‐goat IgG(H+L): #A0181, Beyotime Biotechnology, 1:1000.

### Statistical Analysis and Selection of Representative Images

All statistical analyses were performed using GraphPad Prism 10.0 software. For the in vitro study, all biological replicates using primary cultured cells correspond to independent experiments from distinct expansions and passage numbers, with technical replicates (the exact replicate number was indicated in Figure Legends). As each experimental data set was an average of a large number of cultured cells, the data were assumed normally distributed based on the central limit theorem. The Shapiro‐Wilk test (*P* < 0.05) was used to test the normality of all data obtained from in vivo study. Data with normal distribution were represented as mean ± SEM. For comparisons between two groups, analyses were performed using a two‐tailed Student's *t* test. Data that failed the normality test were analyzed by the Mann‐Whitney U test for two‐group comparisons. Comparisons among multiple groups were made using one‐way analysis of variance (ANOVA) followed by Tukey post hoc multiple comparisons test or two‐way ANOVA followed by Sidak post hoc multiple comparisons test. Raw P values were provided for two‐group comparisons. *P* <0.05 was considered statistically significant.

Representative images were selected based on high quality and accurate representation of similarity with the mean value for each experimental group.

## Conflict of Interest

The authors declare no conflict of interest.

## Author Contributions

Z.H., M.H., and Z.Z. contribute equally to this work and co‐first authors. The conceptualization of the work is carried out by Y.X. and J.R. Methodology is developed by Y.X., J.R., and Z.Z. The investigation is conducted by Z.H., M.H., X.Y., J.Z., and W.W. Visualization is performed by Z.H., M.H., and J.R. Supervision is provided by Y.X., J.R., and Z.Z. The original draft is written by Y.X. and Z.H., while the review and editing are completed by Y.X. and J.R.

## Supporting information



Supporting Information

## Data Availability

The data that support the findings of this study are available from the corresponding author upon reasonable request.
