# Supplementary material for: Loss of Hepatic Angiotensinogen Attenuates Diastolic Dysfunction in Heart Failure with Preserved Ejection Fraction
Source: Adv Sci (Weinh). 2025 Sep 1;12(43):e07554. doi: 10.1002/advs.202507554 (PMC12631940; doi:10.1002/advs.202507554)
Supplement: Supplementary file 1 — Supporting Information [file ADVS-12-e07554-s001.pdf]

## Supporting Information

## Supplemental Figure Legends

Supplemental Figure 1

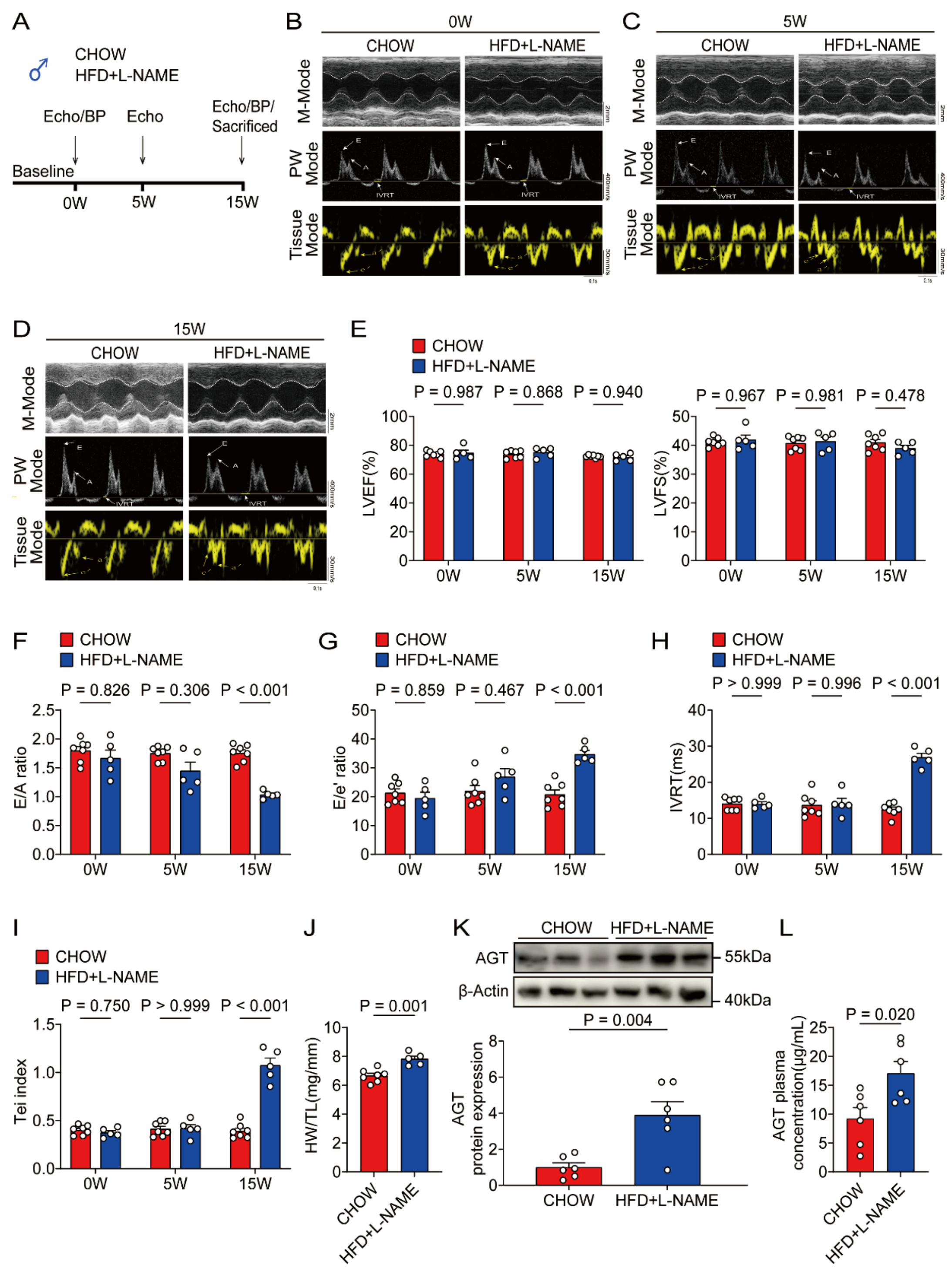


**Supplemental Figure 1. HFpEF increased hepatic AGT abundances and plasma AGT concentrations in male mice.**

A. Experimental workflow and analysis for HFpEF model establishment in male

mice (n=7 for CHOW group, and n=5 for HFD+L-NAME group). B. Representative echocardiography images obtained from male mice at baseline (n=7 for CHOW group, and n=5 for HFD+L-NAME group). C. Representative echocardiography images obtained from male mice fed with a high-fat diet and L-NAME for 5 weeks (n=7 for CHOW group, and n=5 for HFD+L-NAME group). D. Representative echocardiography images obtained from male mice fed with a high-fat diet and L-NAME for 15 weeks (n=7 for CHOW group, and n=5 for HFD+L-NAME group). E. Left ventricular ejection fraction (LVEF%) and left ventricular fraction shortening (LVFS%) were quantified via echocardiography (n=7 for CHOW group, and n=5 for HFD+L-NAME group). F. E/A ratio was quantified via echocardiography (n=7 for CHOW group, and n=5 for HFD+L-NAME group). G. E/e' ratio was quantified via echocardiography (n=7 for CHOW group, and n=5 for HFD+L-NAME group). H. IVRT was quantified via echocardiography (n=7 for CHOW group, and n=5 for HFD+L-NAME group). I. Tei index was quantified via echocardiography (n=7 for CHOW group, and n=5 for HFD+L-NAME group). J. Ratio of heart weight to tibia length (HW/TL) was measured in male mice 15 weeks after feeding with HFD+L-NAME (n=7 for CHOW group, and n=5 for HFD+L-NAME group). K. Western blotting detection and quantification of AGT protein abundances in livers obtained from male mice at 15w after feeding with HFD and L-NAME (n=6 for each group). L. Plasma AGT concentrations in male HFpEF mice were measured by ELISA (n=6 for each group). Two-way ANOVA was used for statistical analysis in E-I, and Student's t test was used for statistical analysis in J-L.

Supplemental Figure 2

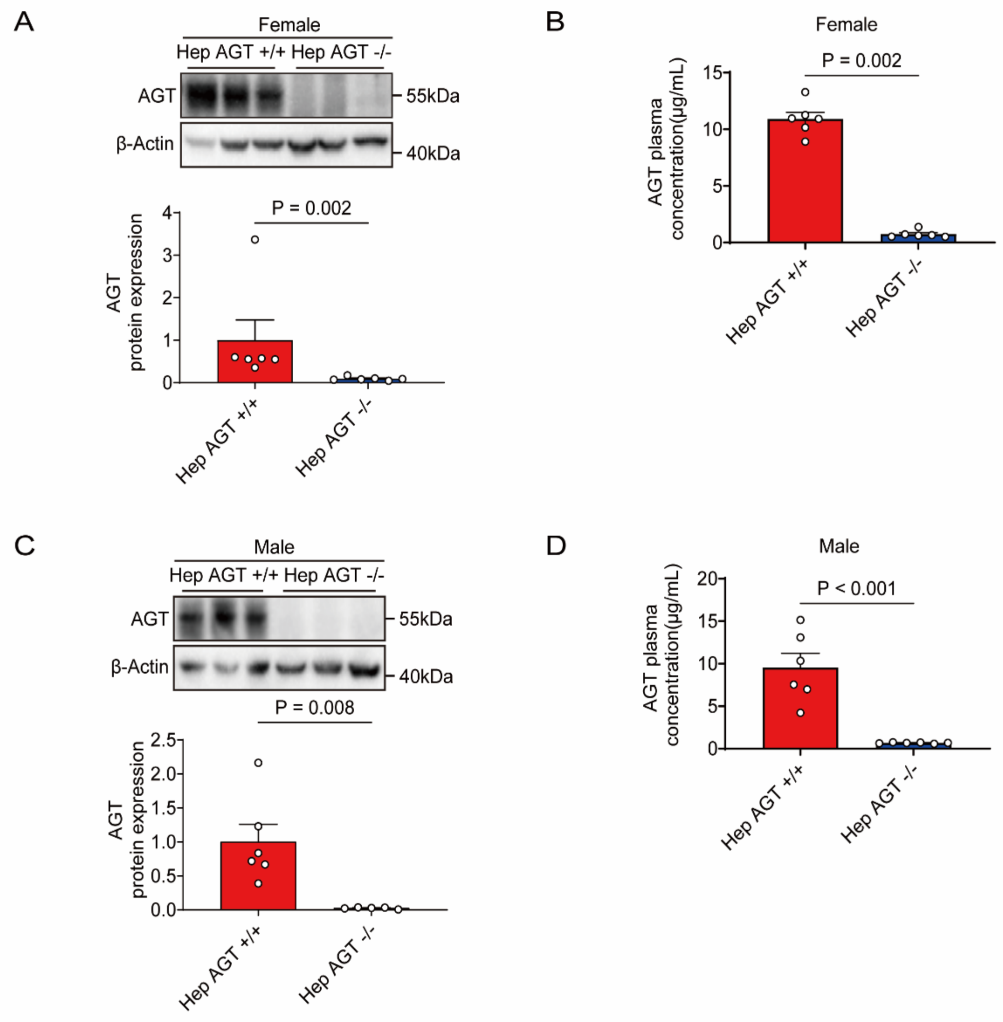


**Supplemental Figure 2. Establishment of hepatic AGT specific deficiency mouse model.**

A. Western blotting detection and quantification of AGT protein abundances in livers obtained from female hepAGT+/+ and female hepAGT-/- mice (n=6 for each group). B. Plasma AGT concentrations in female hepAGT+/+ and female hepAGT-/- mice were measured by ELISA (n=6 for each group). C. Western blotting detection and quantification of AGT protein abundances in livers obtained from male hepAGT+/+ and male hepAGT-/- mice (n=6 for hepAGT +/+ group, and n=5 for hepAGT -/- group). D. Plasma AGT concentrations in male hepAGT+/+ and male hepAGT-/- mice were measured by ELISA (n=6 for each group). Mann-Whitney U test was used for statistical analysis in A and B. Student's t test was used for statistical analysis in C and D.

Supplemental Figure 3

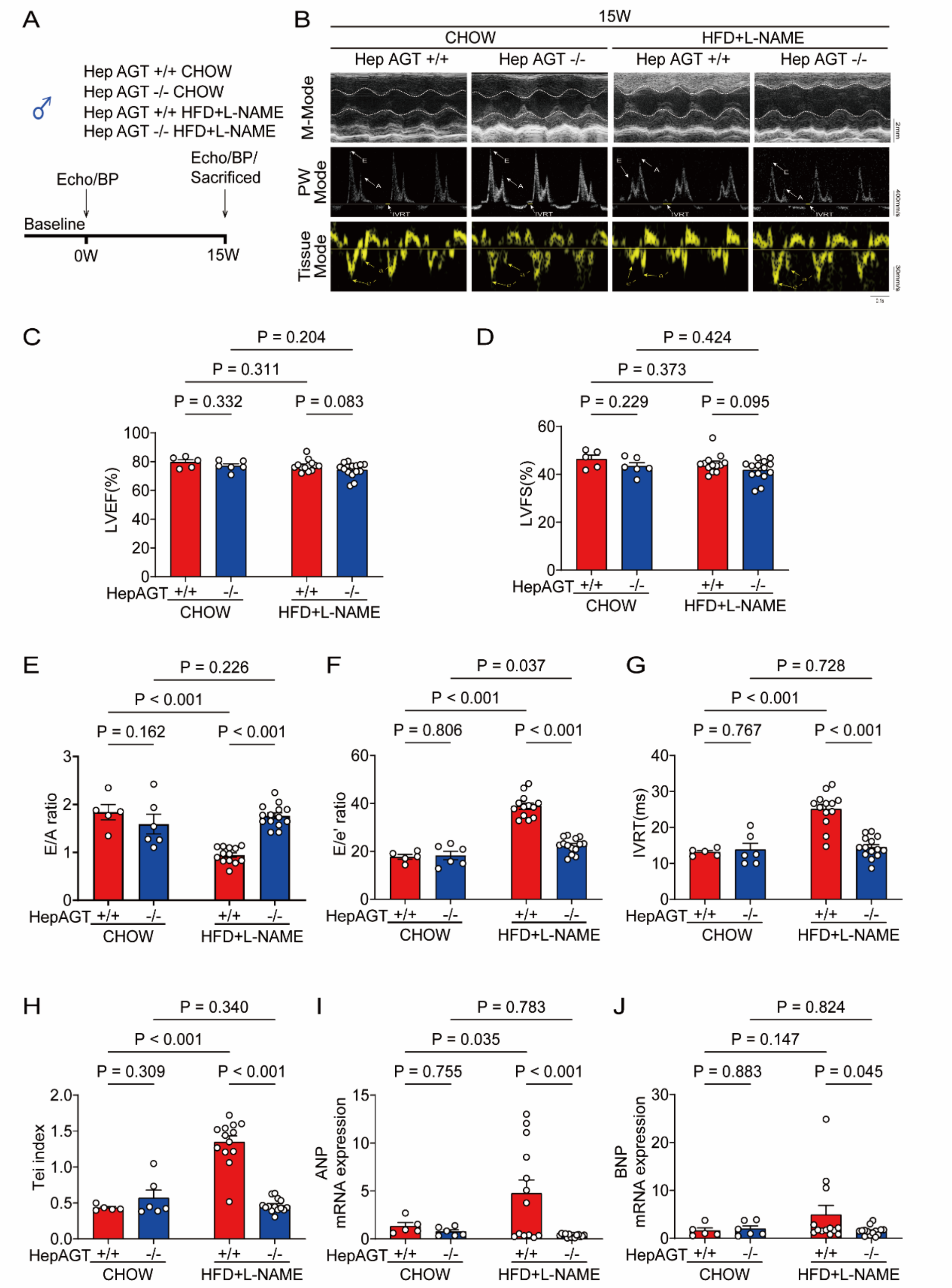


**Supplemental Figure 3. Deficiency of hepatic AGT alleviated diastolic dysfunction in male HFpEF mice.**

A. Experimental workflow and analysis for the effects of hepatic AGT deletion on HFpEF in male mice (n=5 for hepAGT+/+ CHOW group, n=6 for hepAGT-/- CHOW group, n=13 for hepAGT+/+ HFD+L-NAME group, n=14 for hepAGT-/-

HFD+L-NAME group). B. Representative echocardiography images obtained from male mice at 15w after feeding with chow or HFD+L-NAME (n=5 for hepAGT+/+ CHOW group, n=6 for hepAGT-/- CHOW group, n=13 for hepAGT+/+ HFD+L-NAME group, n=14 for hepAGT-/- HFD+L-NAME group). C. Left ventricular ejection fraction (LVEF%) was quantified via echocardiography (n=5 for hepAGT+/+ CHOW group, n=6 for hepAGT-/- CHOW group, n=13 for hepAGT+/+ HFD+L-NAME group, n=14 for hepAGT-/- HFD+L-NAME group). D. Left ventricular fraction shortening (LVFS%) was quantified via echocardiography (n=5 for hepAGT+/+ CHOW group, n=6 for hepAGT-/- CHOW group, n=13 for hepAGT+/+ HFD+L-NAME group, n=14 for hepAGT-/- HFD+L-NAME group). E. E/A ratio was quantified via echocardiography (n=5 for hepAGT+/+ CHOW group, n=6 for hepAGT-/- CHOW group, n=13 for hepAGT+/+ HFD+L-NAME group, n=14 for hepAGT-/- HFD+L-NAME group). F. E/e' ratio was quantified via echocardiography (n=5 for hepAGT+/+ CHOW group, n=6 for hepAGT-/- CHOW group, n=13 for hepAGT+/+ HFD+L-NAME group, n=14 for hepAGT-/- HFD+L-NAME group). G. IVRT was quantified via echocardiography (n=5 for hepAGT+/+ CHOW group, n=6 for hepAGT-/- CHOW group, n=13 for hepAGT+/+ HFD+L-NAME group, n=14 for hepAGT-/- HFD+L-NAME group). H. Tei index was quantified via echocardiography (n=5 for hepAGT+/+ CHOW group, n=6 for hepAGT-/- CHOW group, n=13 for hepAGT+/+ HFD+L-NAME group, n=14 for hepAGT-/- HFD+L-NAME group). I. mRNA abundance of cardiac atrial natriuretic peptide (ANP) was assessed in male HFpEF hepAGT+/+ and male HFpEF hepAGT-/- mice (n=5 for hepAGT+/+ CHOW group, n=6 for hepAGT-/- CHOW group, n=13 for hepAGT+/+ HFD+L-NAME group, n=14 for hepAGT-/- HFD+L-NAME group). J. mRNA abundance of cardiac brain natriuretic peptide (BNP) was assessed in male HFpEF hepAGT+/+ and male HFpEF hepAGT-/- mice (n=5 for hepAGT+/+ CHOW group, n=6 for hepAGT-/- CHOW group, n=13 for hepAGT+/+ HFD+L-NAME group, n=14 for hepAGT-/- HFD+L-NAME group). Two-way ANOVA was used for statistical analysis.

Supplemental Figure 4

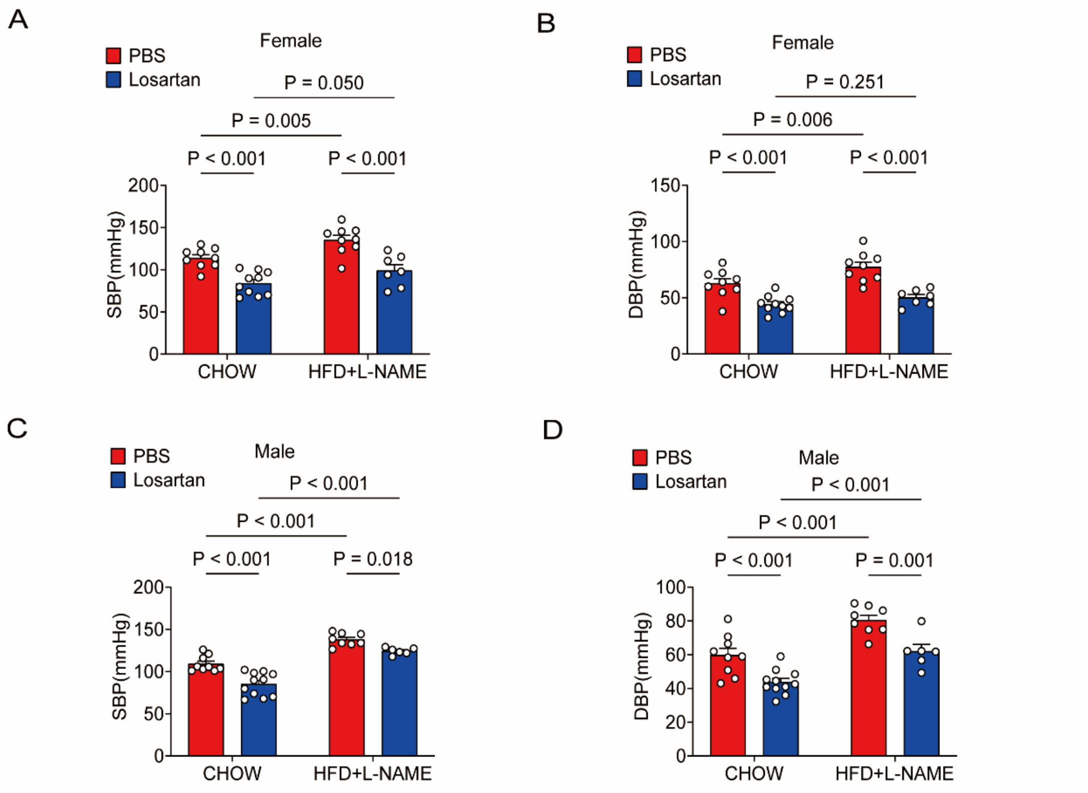


**Supplemental Figure 4. Losartan treatment decreased the blood pressure of HFpEF mice.**

A. Systolic blood pressure was measured in female HFpEF mice feeding with PBS or Losartan (n=9 for PBS CHOW group, n=10 for Losartan CHOW group, n=9 for PBS HFD+L-NAME group, n=7 for Losartan HFD+L-NAME group). B. Diastolic blood pressure was measured in female HFpEF mice feeding with PBS or Losartan (n=9 for PBS CHOW group, n=10 for Losartan CHOW group, n=9 for PBS HFD+L-NAME group, n=7 for Losartan HFD+L-NAME group). C. Systolic blood pressure was measured in male HFpEF mice feeding with PBS or Losartan (n=9 for PBS CHOW group, n=11 for Losartan CHOW group, n=8 for PBS HFD+L-NAME group, n=6 for Losartan HFD+L-NAME group). D. Diastolic blood pressure was measured in male HFpEF mice feeding with PBS or Losartan (n=9 for PBS CHOW group, n=11 for Losartan CHOW group, n=8 for PBS HFD+L-NAME group, n=6 for Losartan HFD+L-NAME group). Two-way ANOVA was used for statistical analysis.

Supplemental Figure 5

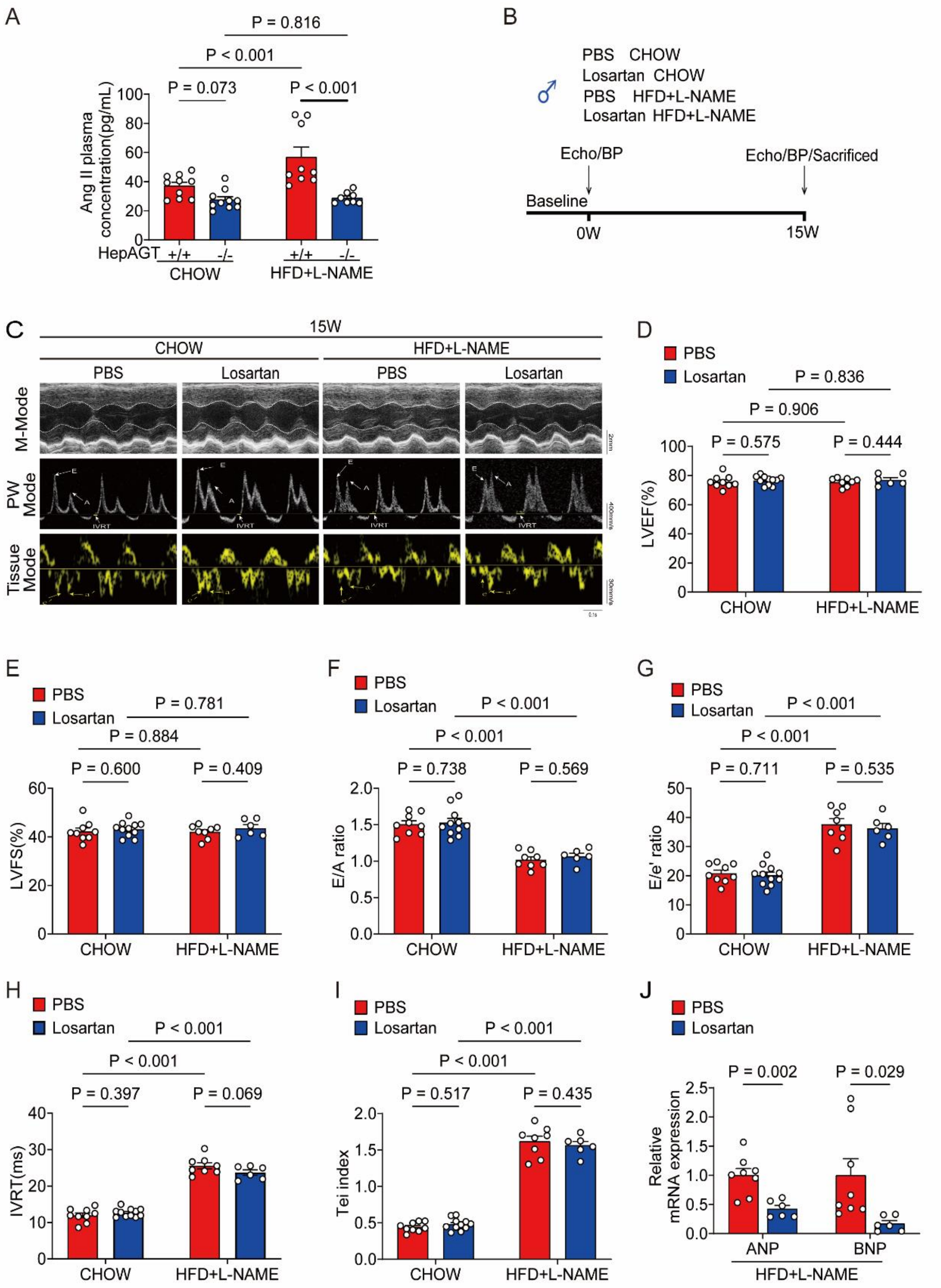


**Supplemental Figure 5. Inhibition of systemic AngII by Losartan exhibited no effects on cardiac diastolic function in male HFpEF mice.**

A. Plasma AngII concentrations in male HFpEF hepAGT+/+ and male HFpEF hepAGT-/- mice were measured by ELISA (n=10 for hepAGT+/+ CHOW group,

n=10 for hepAGT-/- CHOW group, n=9 for hepAGT+/+ HFD+L-NAME group, n=8 for hepAGT-/- HFD+L-NAME group). B. Experimental workflow and analysis for the effects of Losartan treatment on HFpEF in male mice (n=9 for PBS CHOW group, n=11 for Losartan CHOW group, n=8 for PBS HFD+L-NAME group, n=6 for Losartan HFD+L-NAME group). C. Representative echocardiography images obtained from male HFpEF mice at 15w after feeding with PBS or Losartan (n=9 for PBS CHOW group, n=11 for Losartan CHOW group, n=8 for PBS HFD+L-NAME group, n=6 for Losartan HFD+L-NAME group). D. Left ventricular ejection fraction (LVEF%) was quantified via echocardiography (n=9 for PBS CHOW group, n=11 for Losartan CHOW group, n=8 for PBS HFD+L-NAME group, n=6 for Losartan HFD+L-NAME group). E. Left ventricular fraction shortening (LVFS%) was quantified via echocardiography (n=9 for PBS CHOW group, n=11 for Losartan CHOW group, n=8 for PBS HFD+L-NAME group, n=6 for Losartan HFD+L-NAME group). F. E/A ratio was quantified via echocardiography (n=9 for PBS CHOW group, n=11 for Losartan CHOW group, n=8 for PBS HFD+L-NAME group, n=6 for Losartan HFD+L-NAME group). G. E/e' ratio was quantified via echocardiography (n=9 for PBS CHOW group, n=11 for Losartan CHOW group, n=8 for PBS HFD+L-NAME group, n=6 for Losartan HFD+L-NAME group). H. IVRT was quantified via echocardiography (n=9 for PBS CHOW group, n=11 for Losartan CHOW group, n=8 for PBS HFD+L-NAME group, n=6 for Losartan HFD+L-NAME group). I. Tei index was quantified via echocardiography (n=9 for PBS CHOW group, n=11 for Losartan CHOW group, n=8 for PBS HFD+L-NAME group, n=6 for Losartan HFD+L-NAME group). J. mRNA abundances of cardiac atrial natriuretic peptide (ANP) and cardiac brain natriuretic peptide (BNP) were assessed in male HFpEF mice feeding with PBS or Losartan (n=8 for PBS HFD+L-NAME group, n=6 for Losartan HFD+L-NAME group). Two-way ANOVA were used for statistical analysis in A and D-I, and Student's t test was used for statistical analysis in J.

Supplemental Figure 6

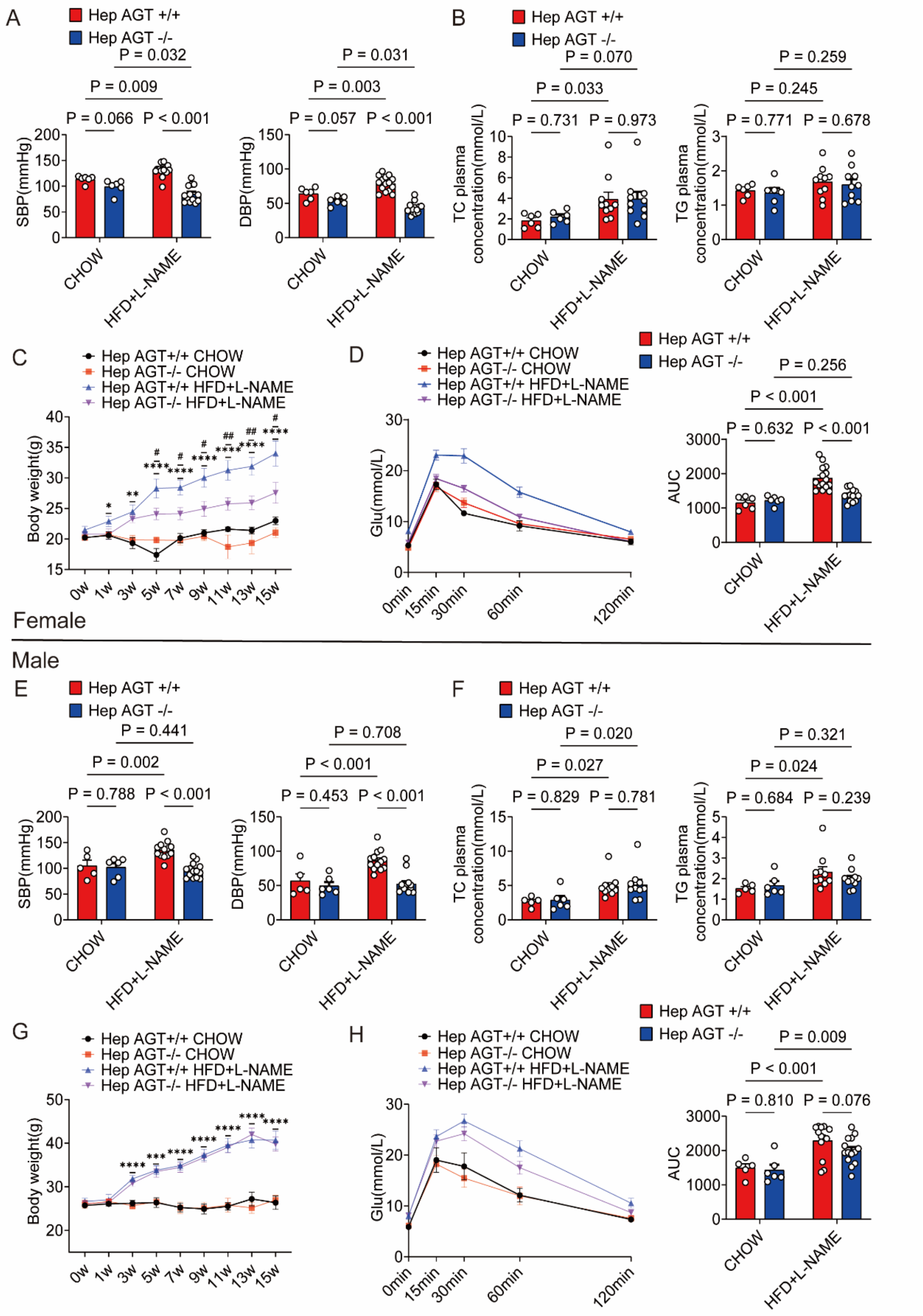


**Supplemental Figure 6. Sex differences in metabolic phenotypes regulated by hepatic AGT in HFpEF mice model.**

A. Systolic and diastolic blood pressure were measured in female hepAGT+/+ and female hepAGT-/- mice at 15w after feeding with Chow or HFD+L-NAME,

respectively (n=6 for hepAGT+/+ CHOW group, n=6 for hepAGT-/- CHOW group, n=15 for hepAGT+/+ HFD+L-NAME group, n=13 for hepAGT-/- HFD+L-NAME group). B. Plasma TC and TG concentrations were measured in female hepAGT+/+ and female hepAGT-/- mice at 15w after feeding with Chow or HFD+L-NAME, respectively (n=6 for hepAGT+/+ CHOW group, n=6 for hepAGT-/- CHOW group, n=10 for hepAGT+/+ HFD+L-NAME group, n=10 for hepAGT-/- HFD+L-NAME group). C. Body weight was measured in female hepAGT+/+ and female hepAGT-/- mice at selected intervals (0w, 1w, 3w, 5w, 7w, 9w, 11w, 13w, 15w) after feeding with Chow or HFD+L-NAME, respectively (n=6 for hepAGT+/+ CHOW group, n=6 for hepAGT-/- CHOW group, n=15 for hepAGT+/+ HFD+L-NAME group, n=13 for hepAGT-/- HFD+L-NAME group). D. Blood glucose levels in IPGTT were measured in female hepAGT+/+ and female hepAGT-/- mice at 15w after feeding with Chow or HFD+L-NAME, respectively (n=6 for hepAGT+/+ CHOW group, n=6 for hepAGT-/- CHOW group, n=15 for hepAGT+/+ HFD+L-NAME group, n=13 for hepAGT-/- HFD+L-NAME group). E. Systolic and diastolic blood pressure were measured in male hepAGT+/+ and male hepAGT-/- mice at 15w after feeding with Chow or HFD+L-NAME, respectively (n=5 for hepAGT+/+ CHOW group, n=6 for hepAGT-/- CHOW group, n=13 for hepAGT+/+ HFD+L-NAME group, n=14 for hepAGT-/- HFD+L-NAME group). F. Plasma TC and TG concentrations were measured in male hepAGT+/+ and male hepAGT-/- mice at 15w after feeding with Chow or HFD+L-NAME, respectively (n=5 for hepAGT+/+ CHOW group, n=6 for hepAGT-/- CHOW group, n=10 for hepAGT+/+ HFD+L-NAME group, n=10 for hepAGT-/- HFD+L-NAME group). G. Body weight was measured in male hepAGT+/+ and male hepAGT-/- mice at selected intervals (0w, 1w, 3w, 5w, 7w, 9w, 11w, 13w, 15w) after feeding with Chow or HFD+L-NAME, respectively (n=5 for hepAGT+/+ CHOW group, n=6 for hepAGT-/- CHOW group, n=13 for hepAGT+/+ HFD+L-NAME group, n=14 for hepAGT-/- HFD+L-NAME group). H. Blood glucose levels in IPGTT were measured in male hepAGT+/+ and male hepAGT-/- mice at 15w after feeding with Chow or HFD+L-NAME, respectively (n=5 for hepAGT+/+ CHOW group, n=6 for hepAGT-/- CHOW group, n=13 for hepAGT+/+ HFD+L-NAME group, n=14 for hepAGT-/- HFD+L-NAME group). Two-way ANOVA was used for statistical

analysis. * P<0.05; ** P<0.005; ***P<0.0005; ****P<0.0001 HepAGT+/+ CHOW vs HepAGT+/+HFD+L-NAME; #P<0.05; ##P<0.005 HepAGT+/+ HFD+L-NAME vs HepAGT-/-HFD+L-NAME.

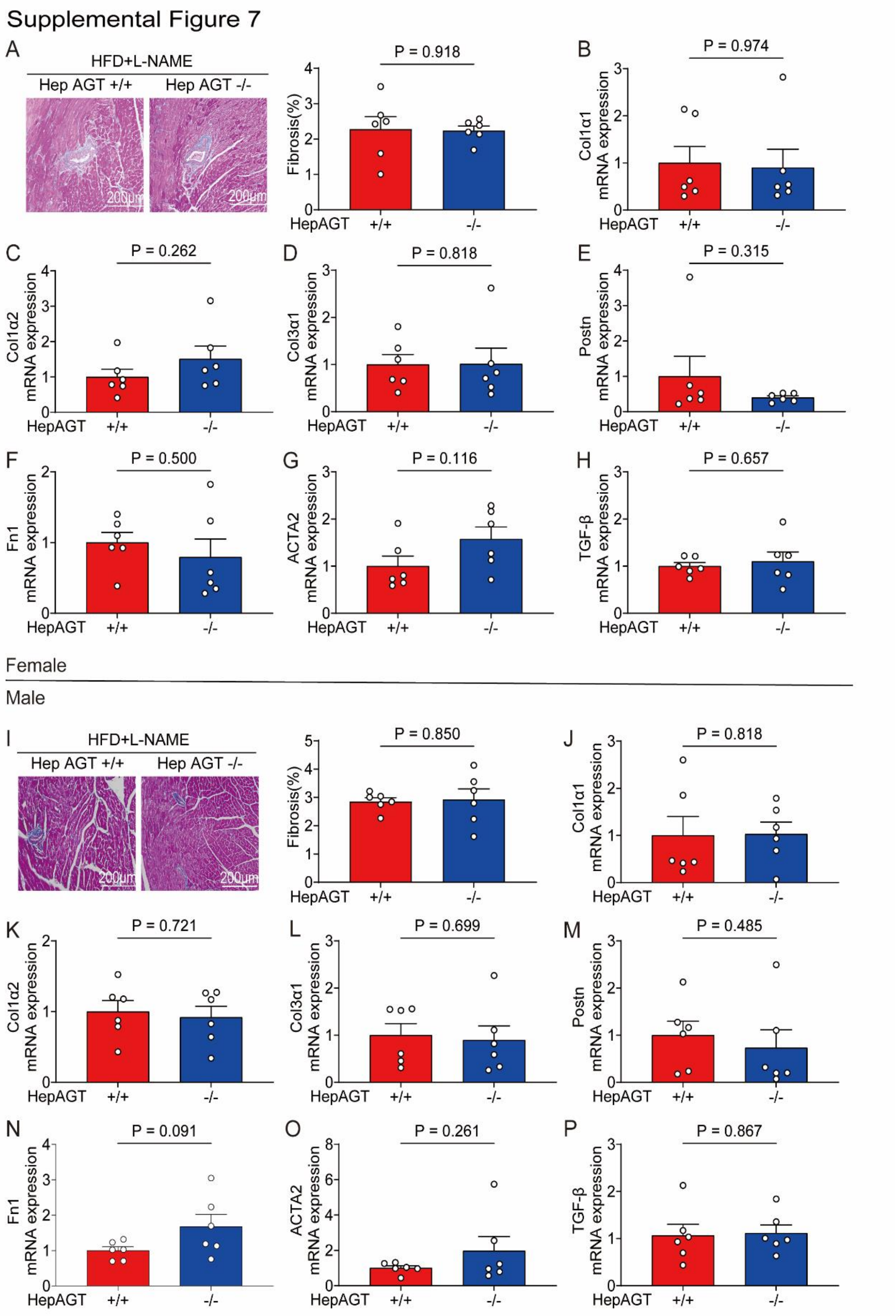

**Supplemental Figure 7. HepAGT depletion exhibited no effects on cardiac fibrosis in HFpEF mice.**

A. Representative Masson trichrome staining images of cardiac tissues obtained from female hepAGT+/+ and female hepAGT-/- mice at 15w after feeding with HFD+L-NAME. Cardiac fibrotic areas were then calculated in female HFpEF hepAGT+/+ and female HFpEF hepAGT-/- mice (n=6 for each group, Bar=200μm). B. mRNA abundance of Col1α1 was assessed in female HFpEF hepAGT+/+ and female HFpEF hepAGT-/- mice (n=6 for each group). C. mRNA abundance of Col1α2 was assessed in female HFpEF hepAGT+/+ and female HFpEF hepAGT-/- mice (n=6 for each group). D. mRNA abundance of Col3α1 was assessed in female HFpEF hepAGT+/+ and female HFpEF hepAGT-/- mice (n=6 for each group). E. mRNA abundance of Postn was assessed in female HFpEF hepAGT+/+ and female HFpEF hepAGT-/- mice (n=6 for each group). F. mRNA abundance of Fn1 was assessed in female HFpEF hepAGT+/+ and female HFpEF hepAGT-/- mice (n=6 for each group). G. mRNA abundance of ACTA2 was assessed in female HFpEF hepAGT+/+ and female HFpEF hepAGT-/- mice (n=6 for each group). H. mRNA abundance of TGFβ was assessed in female HFpEF hepAGT+/+ and female HFpEF hepAGT-/- mice (n=6 for each group). I. Representative Masson trichrome staining images of cardiac tissues obtained from male hepAGT+/+ and male hepAGT-/- mice at 15w after feeding with HFD+L-NAME. Cardiac fibrotic areas were then calculated in male HFpEF hepAGT+/+ and male HFpEF hepAGT-/- mice (n=6 for each group, Bar=200μm). J. mRNA abundance of Col1α1 was assessed in male HFpEF hepAGT+/+ and male HFpEF hepAGT-/- mice (n=6 for each group). K. mRNA abundance of Col1α2 was assessed in male HFpEF hepAGT+/+ and male HFpEF hepAGT-/- mice (n=6 for each group). L. mRNA abundance of Col3α1 was assessed in male HFpEF hepAGT+/+ and male HFpEF hepAGT-/- mice (n=6 for each group). M. mRNA abundance of Postn was assessed in male HFpEF hepAGT+/+ and male HFpEF hepAGT-/- mice (n=6 for each group). N. mRNA abundance of Fn1 was assessed in male HFpEF hepAGT+/+ and male HFpEF hepAGT-/- mice (n=6 for each group). O. mRNA abundance of ACTA2 was assessed in male HFpEF hepAGT+/+ and male HFpEF hepAGT-/- mice (n=6 for each group). P. mRNA abundance of

TGFβ was assessed in male HFpEF hepAGT+/+ and male HFpEF hepAGT-/- mice (n=6 for each group). Student's t test was used for statistical analysis in A, C, E-I, K, L, N, O, P. Mann-Whitney U test was used for statistical analysis in B, D, J, M.

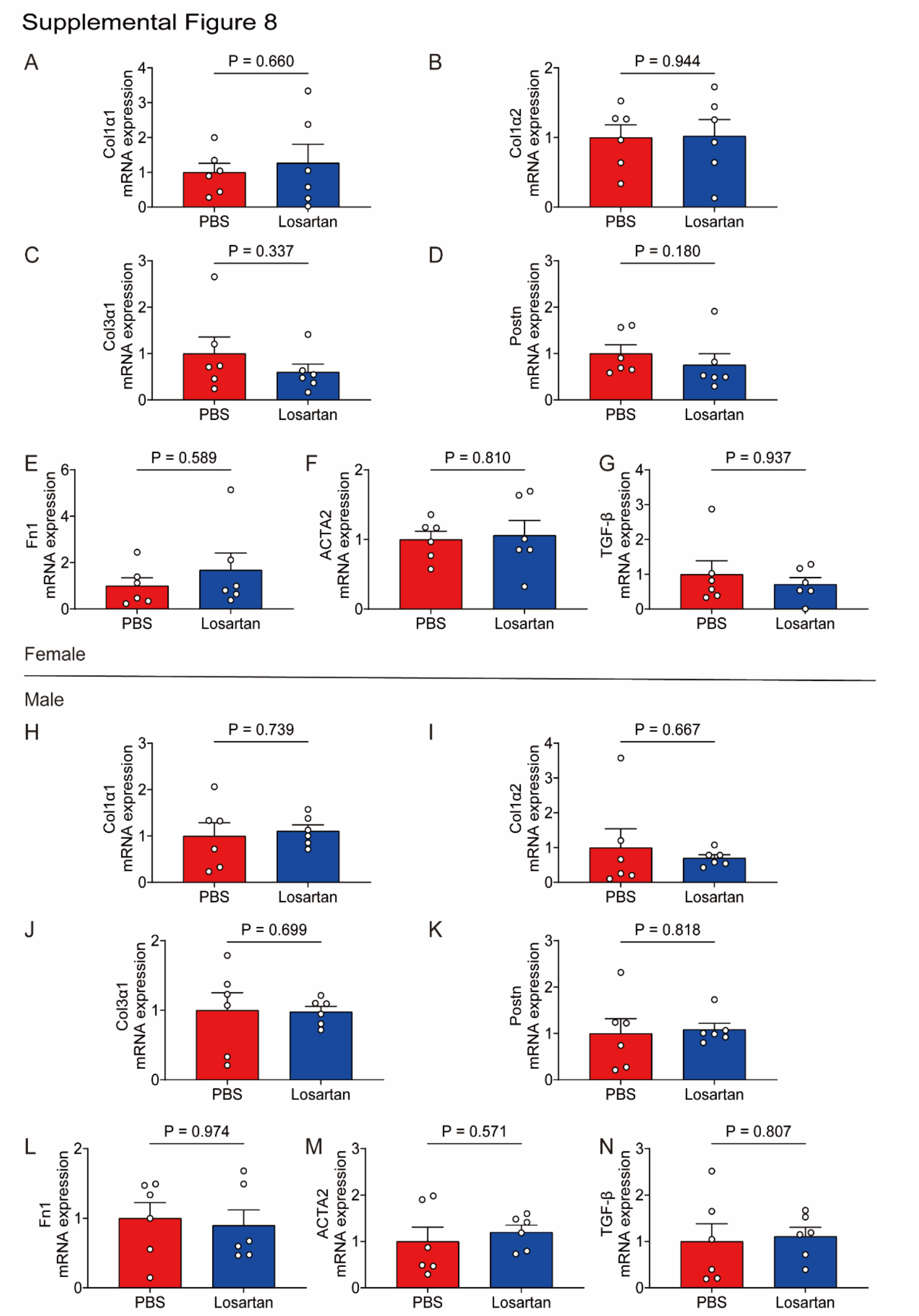


**Supplemental Figure 8. Losartan treatment exhibited no effects on cardiac fibrosis in HFpEF mice.**

A. mRNA abundance of Col1α1 was assessed in female HFpEF hepAGT+/+ mice treated with losartan and those treated with PBS (n=6 for each group). B.

mRNA abundance of Col1α2 was assessed in female HFpEF hepAGT+/+ mice treated with losartan and those treated with PBS (n=6 for each group). C. mRNA abundance of Col3α1 was assessed in female HFpEF hepAGT+/+ mice treated with losartan and those treated with PBS (n=6 for each group). D. mRNA abundance of Postn was assessed in female HFpEF hepAGT+/+ mice treated with losartan and those treated with PBS (n=6 for each group). E. mRNA abundance of Fn1 was assessed in female HFpEF hepAGT+/+ mice treated with losartan and those treated with PBS (n=6 for each group). F. mRNA abundance of ACTA2 was assessed in female HFpEF hepAGT+/+ mice treated with losartan and those treated with PBS (n=6 for each group). G. mRNA abundance of TGF-β was assessed in female HFpEF hepAGT+/+ mice treated with losartan and those treated with PBS (n=6 for each group). H. mRNA abundance of Col1α1 was assessed in male HFpEF hepAGT+/+ mice treated with losartan and those treated with PBS (n=6 for each group). I. mRNA abundance of Col1α2 was assessed in male HFpEF hepAGT+/+ mice treated with losartan and those treated with PBS (n=6 for each group). J. mRNA abundance of Col3α1 was assessed in male HFpEF hepAGT+/+ mice treated with losartan and those treated with PBS (n=6 for each group). K. mRNA abundance of Postn was assessed in male HFpEF hepAGT+/+ mice treated with losartan and those treated with PBS (n=6 for each group). L. mRNA abundance of Fn1 was assessed in male HFpEF hepAGT+/+ mice treated with losartan and those treated with PBS (n=6 for each group). M. mRNA abundance of ACTA2 was assessed in male HFpEF hepAGT+/+ mice treated with losartan and those treated with PBS (n=6 for each group). N. mRNA abundance of TGF-β was assessed in male HFpEF hepAGT+/+ mice treated with losartan and those treated with PBS (n=6 for each group). Student's t test was used for statistical analysis in A-C, F, H, M, N. Mann-Whitney U test was used for statistical analysis in D, E, G, I-L.

Supplemental Figure 9

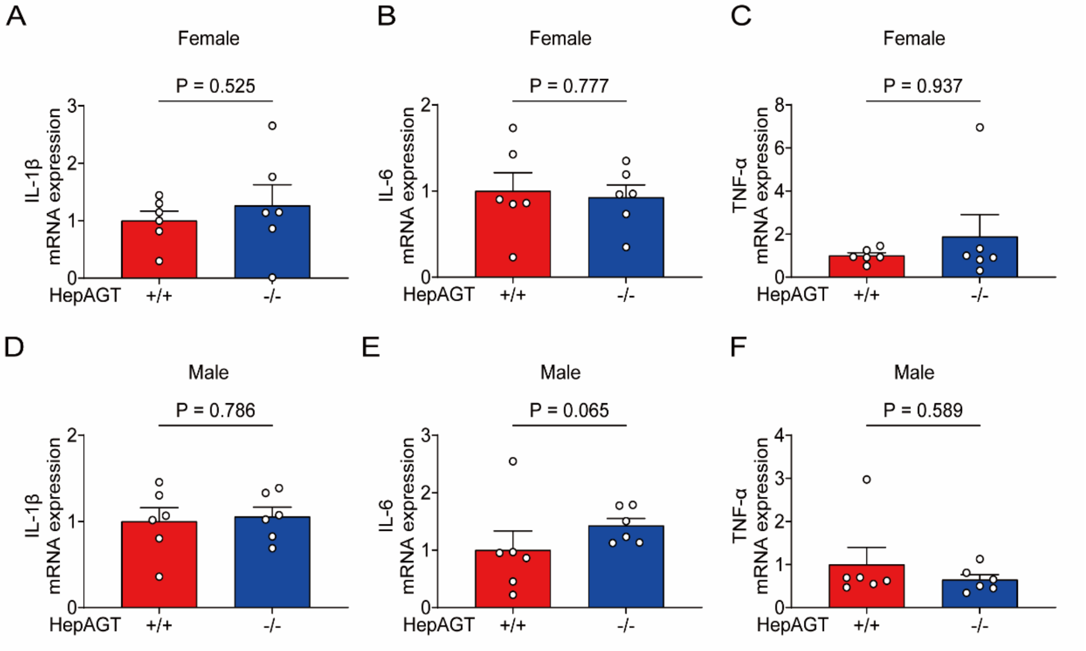


**Supplemental Figure 9. HepAGT depletion exhibited no effects on cardiac inflammation in HFpEF mice.**

A. mRNA abundance of IL-1β was assessed in female HFpEF hepAGT+/+ and female HFpEF hepAGT-/- mice (n=6 for each group). B. mRNA abundance of IL-6 was assessed in female HFpEF hepAGT+/+ and female HFpEF hepAGT-/- mice (n=6 for each group). C. mRNA abundance of TNF-α was assessed in female HFpEF hepAGT+/+ and female HFpEF hepAGT-/- mice (n=6 for each group). D. mRNA abundance of IL-1β was assessed in male HFpEF hepAGT+/+ and male HFpEF hepAGT-/- mice (n=6 for each group). E. mRNA abundance of IL-6 was assessed in male HFpEF hepAGT+/+ and male HFpEF hepAGT-/- mice (n=6 for each group). F. mRNA abundance of TNF-α was assessed in male HFpEF hepAGT+/+ and male HFpEF hepAGT-/- mice (n=6 for each group). Student's t test was used for statistical analysis in A, B, D. Mann-Whitney U test was used for statistical analysis in C, E, F.

Supplemental Figure 10

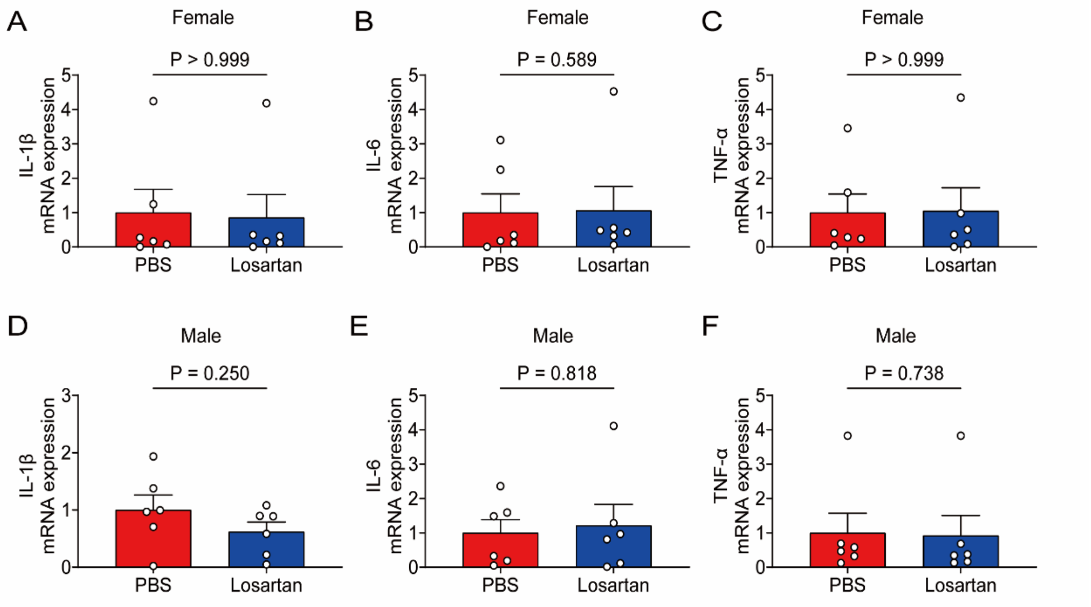


**Supplemental Figure 10. Losartan treatment exhibited no effects on cardiac inflammation in HFpEF mice.**

A. mRNA abundance of IL-1β was assessed in female HFpEF hepAGT+/+ mice treated with losartan and those treated with PBS (n=6 for each group). B. mRNA abundance of IL-6 was assessed in female HFpEF hepAGT+/+ mice treated with losartan and those treated with PBS (n=6 for each group). C. mRNA abundance of TNF-α was assessed in female HFpEF hepAGT+/+ mice treated with losartan and those treated with PBS (n=6 for each group). D. mRNA abundance of IL-1β was assessed in male HFpEF hepAGT+/+ mice treated with losartan and those treated with PBS (n=6 for each group). E. mRNA abundance of IL-6 was assessed in male HFpEF hepAGT+/+ mice treated with losartan and those treated with PBS (n=6 for each group). F. mRNA abundance of TNF-α was assessed in male HFpEF hepAGT+/+ mice treated with losartan and those treated with PBS (n=6 for each group). Student's t test was used for statistical analysis in D. Mann-Whitney U test was used for statistical analysis in A-C, E, F.

Supplemental Figure 11

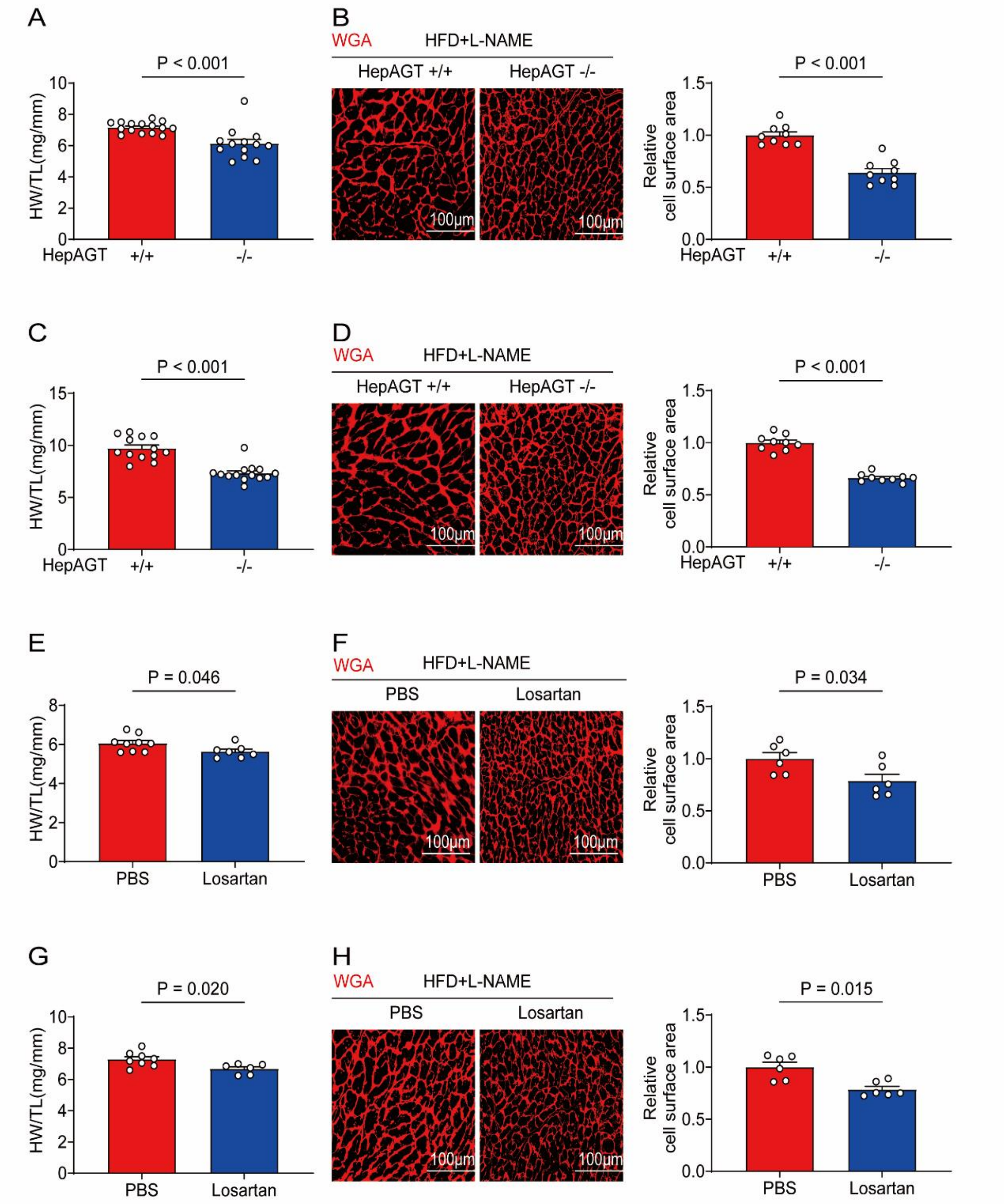


**Supplemental Figure 11. Either deficiency of hepatic AGT or Losartan treatment attenuated myocardial hypertrophy in HFpEF mice.**

A. Ratio of heart weight to tibia length (HW/TL) was measured in female HFpEF hepAGT+/+ and female HFpEF hepAGT-/- mice (n=15 for hepAGT+/+ HFD+L-NAME group, n=13 for hepAGT-/- HFD+L-NAME group). B. Representative WGA staining images of cardiac tissues obtained from female hepAGT+/+ and female hepAGT-/- mice at 15w after feeding with HFD+L-NAME.

Cardiomyocytes areas were then calculated in female HFpEF hepAGT+/+ and female HFpEF hepAGT-/- mice (n=9 for each group, Bar=100μm). C. Ratio of heart weight to tibia length (HW/TL) was measured in male HFpEF hepAGT+/+ and male HFpEF hepAGT-/- mice (n=13 for hepAGT+/+ HFD+L-NAME group, n=14 for hepAGT-/- HFD+L-NAME group). D. Representative WGA staining images of cardiac tissues obtained from male hepAGT+/+ and male hepAGT-/- mice at 15w after feeding with HFD+L-NAME. Cardiomyocytes areas were then calculated in male HFpEF hepAGT+/+ and male HFpEF hepAGT-/- mice (n=9 for each group, Bar=100μm). E. Ratio of heart weight to tibia length (HW/TL) was measured in female HFpEF mice fed with PBS or Losartan (n=9 for PBS HFD+L-NAME group, n=7 for Losartan HFD+L-NAME group). F. Representative WGA staining images of cardiac tissues obtained from female HFpEF mice at 15w after feeding with PBS or Losartan. Cardiomyocytes areas were then calculated in female HFpEF mice fed with PBS or Losartan (n=6 for each group, Bar=100μm). G. Ratio of heart weight to tibia length (HW/TL) was measured in male HFpEF mice fed with PBS or Losartan (n=8 for PBS HFD+L-NAME group, n=6 for Losartan HFD+L-NAME group). H. Representative WGA staining images of cardiac tissues obtained from male HFpEF mice at 15w after feeding with PBS or Losartan. Cardiomyocytes areas were then calculated in male HFpEF mice fed with PBS or Losartan (n=6 for each group, Bar=100μm). Student's t test was used for statistical analysis.

Supplemental Figure 12

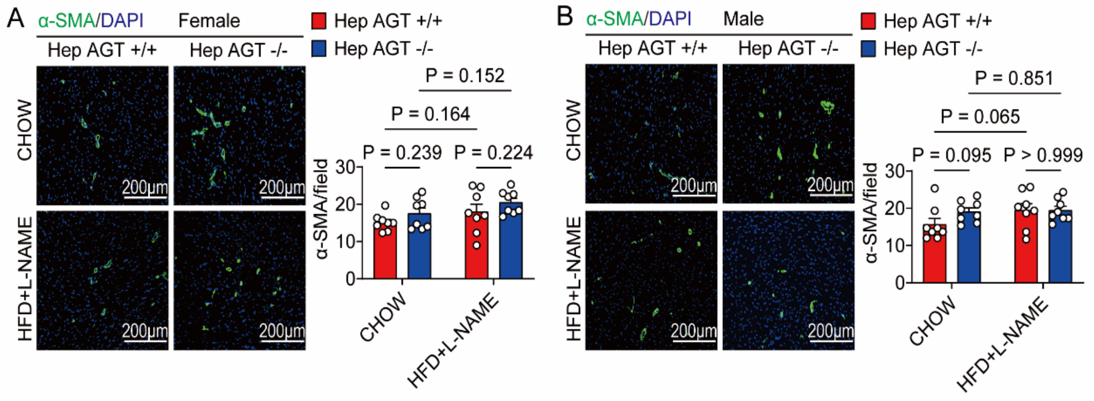


**Supplemental Figure 12. Deficiency of hepatic AGT did not alter myocardial macrovascular density.**

A. Representative α-SMA immune-staining images of cardiac tissues obtained from female hepAGT+/+ and female hepAGT-/- mice at 15w after feeding with CHOW or HFD+L-NAME.α-SMA density of cardiac tissues was then calculated in female HFpEF hepAGT+/+ and female HFpEF hepAGT-/- mice (n=8 for each group, Bar=200μm). B. Representative α-SMA immune-staining images of cardiac tissues obtained from male hepAGT+/+ and male hepAGT-/- mice at 15w after feeding with CHOW or HFD+L-NAME. α-SMA density of cardiac tissues was then calculated in male HFpEF hepAGT+/+ and male HFpEF hepAGT-/- mice (n=8 for each group, Bar=200μm). Two-way ANOVA was used for statistical analysis.

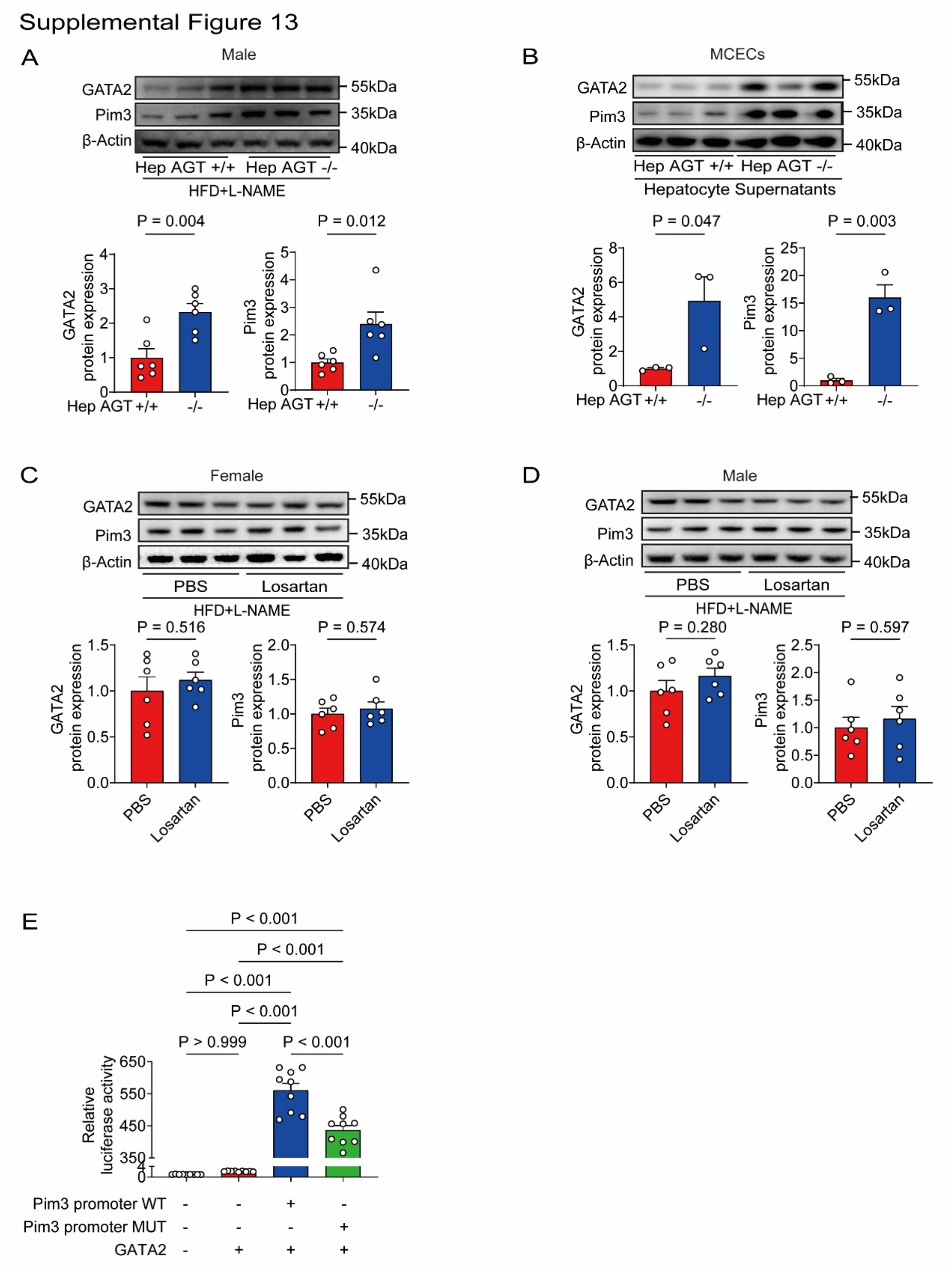


**Supplemental Figure 13. Deficiency of hepatic AGT promoted cardiac microvascular angiogenesis by activating GATA2/Pim3 pathway, which was AngII independent.**

A. Western blotting detection and quantification of GATA2 and Pim3 protein abundances in hearts obtained from male HFpEF hepAGT+/+ and male HFpEF hepAGT-/- mice (n=6 for each group). B. Western blotting detection and

quantification of GATA2 and Pim3 protein abundances in MCECs incubated with hepatocyte supernatants derived from hepAGT+/+ or hepAGT-/- mice (n=3 for each group). C. Western blotting detection and quantification of GATA2 and Pim3 protein abundances in hearts obtained from female HFpEF hepAGT+/+ mice treated with losartan and those treated with PBS (n=6 for each group). D. Western blotting detection and quantification of GATA2 and Pim3 protein abundances in hearts obtained from male HFpEF hepAGT+/+ mice treated with losartan and those treated with PBS (n=6 for each group). E. Dual-Luciferase Reporter assay revealed that GATA2 can bind to the promoter region of Pim3 (n=9 for each group). Student's t test was used for statistical analysis in A-D. One-way ANOVA was used for statistical analysis in E.

Supplemental Figure 14

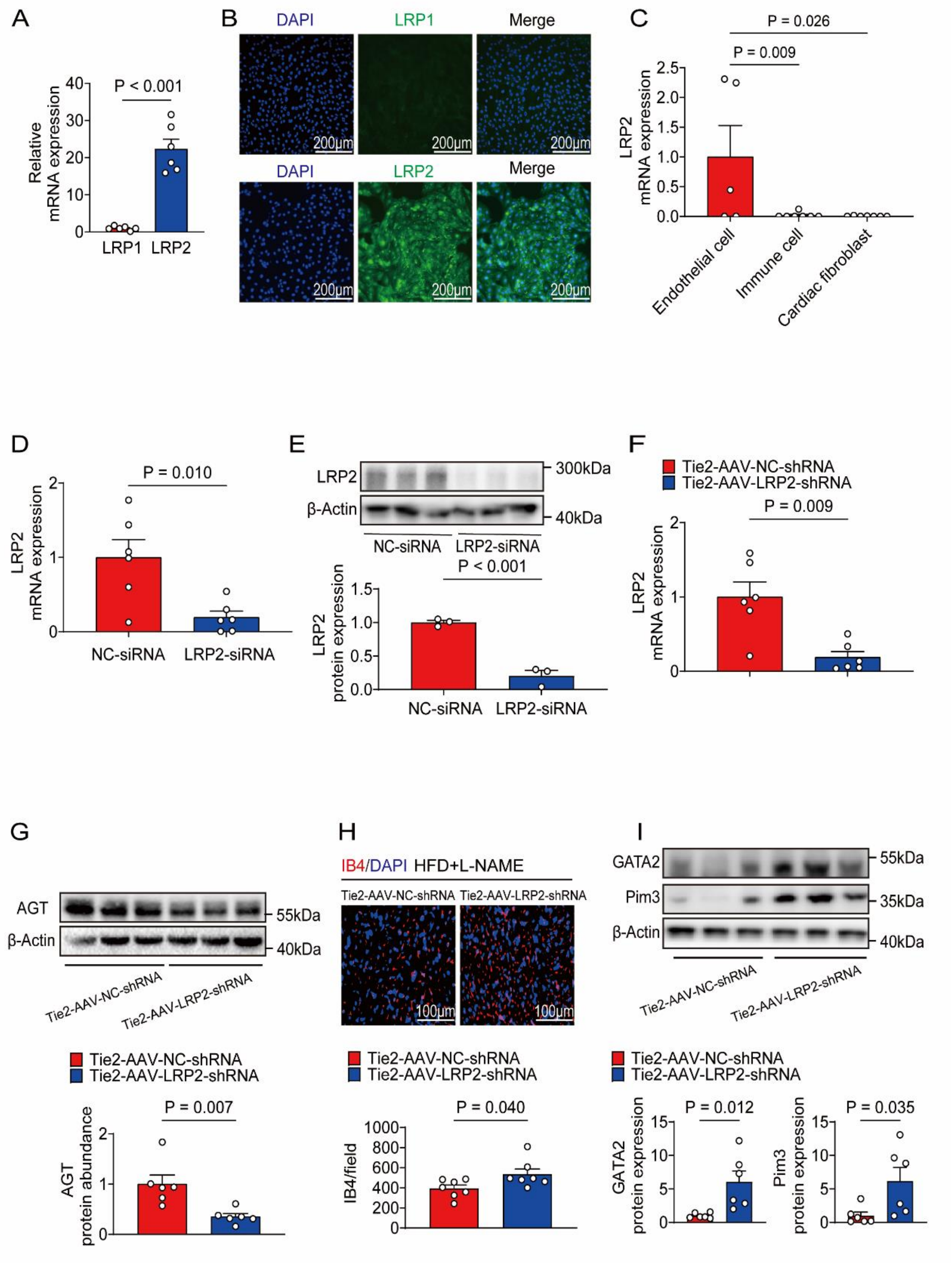


**Supplemental Figure 14. LRP2 was required for hepatocyte-derived AGT internalization in cardiac endothelial cell.**

A. Transcriptional levels of LRP1 and LRP2 in MCECs (n=6 for each group). B. Immunofluorescence staining of LRP1 and LRP2 in MCECs (n=3 for each group, Bar=200μm). C. mRNA abundance of LRP2 was assessed in endothelial

cells, immune cells and cardiac fibroblasts isolated from cardiac tissues, respectively (n=5 for endothelial cell, n=7 for immune cell, and n=7 for cardiac fibroblast). D. mRNA abundance of LRP2 was assessed in MCECs transfected with LRP2 siRNA (n=6 for each group). E. Protein abundance of LRP2 was assessed in MCECs transfected with LRP2 siRNA (n=3 for each group). F. mRNA abundance of LRP2 was assessed in cardiac endothelial cells isolated from female HFpEF mice cardiac tissues injected with Tie2-AAV-LRP2-shRNA (n=6 for each group). G. Protein abundance of AGT was assessed in hearts obtained from female HFpEF mice myocardial injected with Tie2-AAV-LRP2-shRNA (n=6 for each group). H. Representative IB4 immune-staining images and calculation of IB4 density of cardiac tissues were obtained from female HFpEF mice myocardial injected with Tie2-AAV-LRP2-shRNA (n=7 for each group, Bar=100μm). I. Protein abundances of Pim3 and GATA2 were assessed in hearts obtained from female HFpEF mice myocardial injected with Tie2-AAV-LRP2-shRNA (n=6 for each group). Student's t test was used for statistical analysis in A, D, G-I. One-way ANOVA was used for statistical analysis in C. Mann-Whitney U test was used for statistical analysis in E, F.

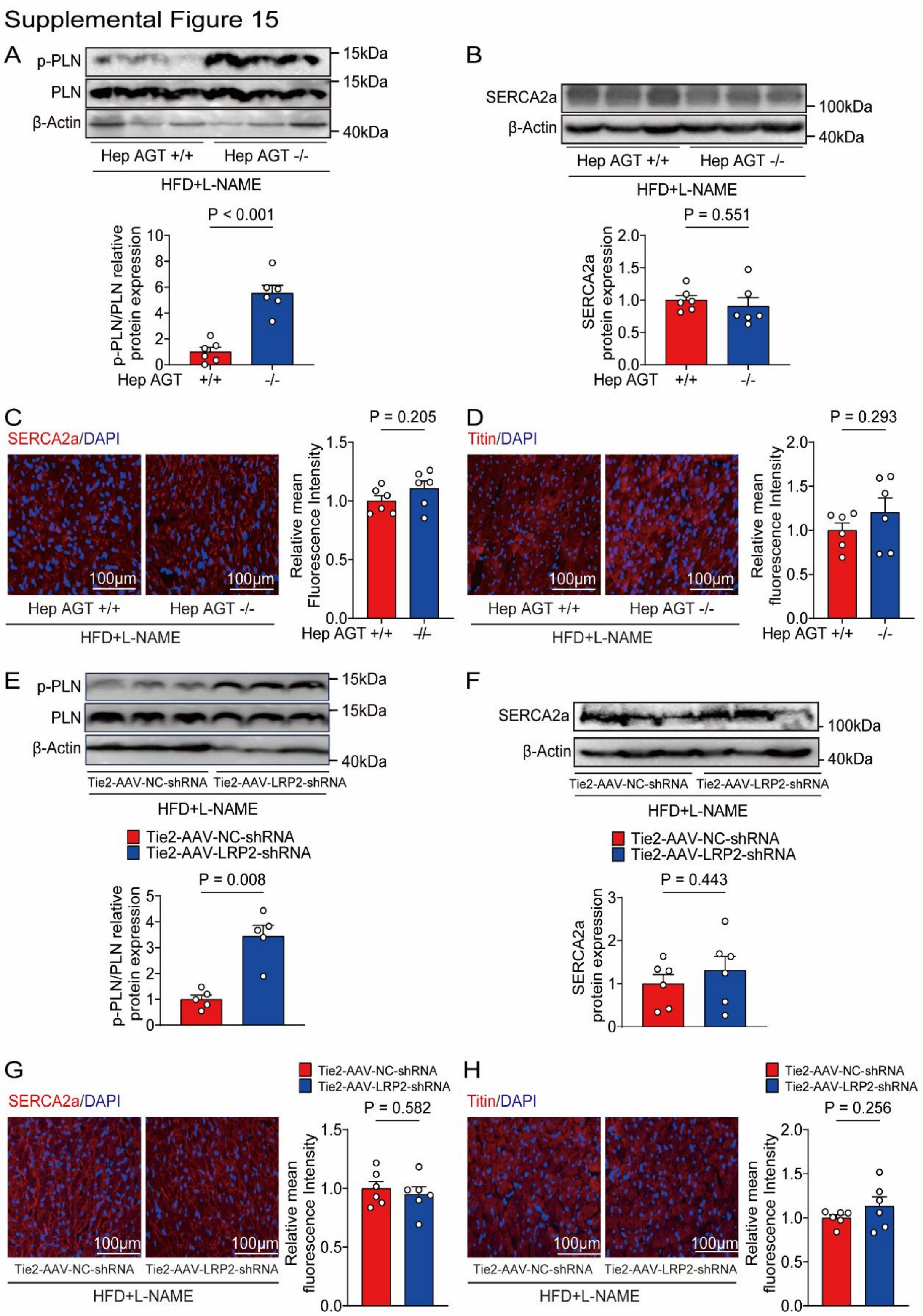


**Supplemental Figure 15. Calcium handling pathway in female HFpEF hepAGT+/+ mice, HFpEF hepAGT-/- mice, HFpEF hepAGT+/+ mice administered Tie2-AAV-NC-shRNA, and HFpEF hepAGT+/+ mice administered Tie2-AAV-LRP2-shRNA.** A. Western blotting detection and

quantification of p-PLN/PLN ratio in hearts obtained from female HFpEF hepAGT+/+ mice and HFpEF hepAGT-/- mice (n=6 for each group). B. Western blotting detection and quantification of SERCA2a protein abundances in hearts obtained from female HFpEF hepAGT+/+ mice and HFpEF hepAGT-/- mice (n=6 for each group). C. Immunofluorescence detection and relative mean fluorescence intensity quantification of SERCA2a in hearts obtained from female HFpEF hepAGT+/+ mice and HFpEF hepAGT-/- mice (n=6 for each group, Bar=100μm). D. Immunofluorescence detection and relative mean fluorescence intensity quantification of Titin in hearts obtained from female HFpEF hepAGT+/+ mice and HFpEF hepAGT-/- mice (n=6 for each group, Bar=100μm). E. Western blotting detection and quantification of p-PLN/PLN ratio in hearts obtained from female HFpEF hepAGT+/+ mice administered Tie2-AAV-NC-shRNA and HFpEF hepAGT+/+ mice administered Tie2-AAV-LRP2-shRNA (n=5 for each group). F. Western blotting detection and quantification of SERCA2a protein abundances in hearts obtained from female HFpEF hepAGT+/+ mice administered Tie2-AAV-NC-shRNA and HFpEF hepAGT+/+ mice administered Tie2-AAV-LRP2-shRNA (n=6 for each group). G. Immunofluorescence detection and relative mean fluorescence intensity quantification of SERCA2a in hearts obtained from female HFpEF hepAGT+/+ mice administered Tie2-AAV-NC-shRNA and HFpEF hepAGT+/+ mice administered Tie2-AAV-LRP2-shRNA (n=6 for each group, Bar=100μm). H. Immunofluorescence detection and relative mean fluorescence intensity quantification of Titin in hearts obtained from female HFpEF hepAGT+/+ mice administered Tie2-AAV-NC-shRNA and HFpEF hepAGT+/+ mice administered Tie2-AAV-LRP2-shRNA (n=6 for each group, Bar=100μm). Student's t test was used for statistical analysis.

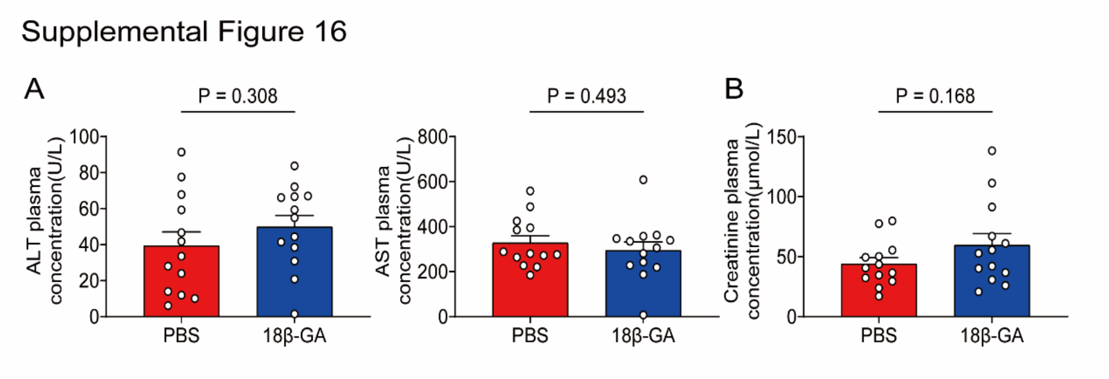


**Supplemental Figure 16. 18β-glycyrrhetinic acid (18β-GA) treatment did not alter hepatic and renal function in HFpEF mice.**

A. Plasma Alanine aminotransferase (ALT) and Aspartate aminotransferase (AST) concentrations were measured in female HFpEF mice treated with 18β-GA (n=13 for each group). B. Plasma creatinine concentrations were measured in female HFpEF mice treated with 18β-GA (n=13 for each group). Student's t test was used for statistical analysis.
